# Effects of Active Video Games on Cardiometabolic Health in Children and Adolescents: Systematic Review and Meta-Analysis of Randomized Controlled Trials

**DOI:** 10.2196/101833

**Published:** 2026-10-13

**Authors:** Wenqi Liu, Yuxin Wang, Tianle Wang, Chunji Zhao, Jun Li, Weixin Chen, Fanhao Meng

**Affiliations:** 1 School of Arts, Beijing Sport University, No.48, Xinxi Road, Haidian District, Beijing, China, Beijing, Beijing, China; 2 School of Physical Education, Shaanxi Normal University, No. 620, West Chang’an Street, Chang’an District, Xi'an, Shaanxi, China; 3 University of Freiburg, Germany, Baden-Wurttemberg, Germany

**Keywords:** active video games, digital health, children, adolescents, cardiometabolic health, physical activity

## Abstract

**Background:**

Cardiometabolic risk factors emerging during childhood and adolescence may persist into adulthood and increase the risk of cardiovascular disease. Although physical activity improves cardiometabolic health, maintaining regular participation remains challenging. Active video games (AVG), which combine physical activity with video game enjoyment, may provide an engaging approach to promote physical activity participation.

**Objective:**

This study aims to evaluate the effects of AVG on cardiometabolic health in children and adolescents and to explore potential moderators of these effects.

**Methods:**

PubMed, Web of Science, the Cochrane Library, and Embase were searched from database inception to July 2026. Eligible randomized controlled trials (RCTs) evaluating AVG in children and adolescents aged 4‐18 years were included, with comparisons against no intervention, sedentary video games, or regular physical activity. Random-effects meta-analyses were conducted to estimate pooled effects. Subgroup analyses explored potential moderators. Risk of bias was assessed using the Cochrane Risk of Bias 2 tool (RoB 2; Cochrane), sensitivity analyses examined robustness, and small-study effects were evaluated when feasible. Certainty of evidence was assessed using GRADE (Grading of Recommendations Assessment, Development and Evaluation).

**Results:**

A total of 14 RCTs involving 942 children and adolescents were included. AVG were associated with a small but significant reduction in BMI (Hedges *g*=−0.23, 95% CI −0.35 to −0.10; 95% prediction interval [PI] −0.43 to −0.01; *P*=.002). SBP (Hedges *g*=−0.18, 95% CI −0.36 to −0.001; 95% PI −0.43 to 0.07; *P*=.0497) showed a small statistically significant reduction, although the 95% PI crossed the null. No significant effects were observed for diastolic blood pressure (DBP) (Hedges *g*=−0.11, 95% CI −0.86 to 0.63; 95% PI −2.09 to 1.75; *P*=.59), high-density lipoprotein cholesterol (HDL) (Hedges *g*=0.05; 95% CI −0.21 to 0.31; 95% PI −0.54 to 0.57; *P*=.65), low-density lipoprotein cholesterol (LDL) (Hedges *g*=−0.26, 95% CI −0.64 to 0.13; 95% PI −1.18 to 0.69; *P*=.15), total cholesterol (TC) (Hedges *g*=−0.96, 95% CI −3.79 to 1.88; 95% PI −5.71 to 3.86; *P*=.37), triglycerides (Hedges *g*=−0.20, 95% CI −0.70 to 0.31; 95% PI −1.41 to 0.99; *P*=.38), heart rate (HR) (Hedges *g*=−0.14, 95% CI −1.74 to 1.46; 95% PI −4.23 to 5.38; *P*=.47), or glucose (Hedges *g*=−0.17, 95% CI −0.37 to 0.02; 95% PI −0.42 to 0.07; *P*=.07) following AVG. No significant BMI subgroup differences were observed by gender, intervention type, or nutritional status.

**Conclusions:**

This review extends previous evidence by providing an updated RCT-only evaluation of a broader pediatric population and a wider range of cardiometabolic outcomes. AVG were associated with small reductions in BMI and systolic blood pressure (SBP), although evidence for other cardiometabolic outcomes remains uncertain. By clarifying both potential benefits and remaining evidence gaps, this review provides a broader basis for future research and intervention development. AVG may serve as an engaging adjunct to support physical activity participation among children and adolescents.

## Introduction

### Rationale

Insufficient physical activity and prolonged sedentary behavior are considered important adverse factors affecting cardiometabolic health in children and adolescents [1-3], and may be associated with reduced insulin sensitivity, abnormalities in glucose and lipid metabolism, elevated blood pressure, and an increased risk of obesity [4-6]. Existing evidence indicates that higher levels of physical activity are associated with lower cardiometabolic risk in children and adolescents [7]. In addition, childhood overweight and obesity warrant particular attention, as children who are overweight or obese are more likely to have abnormal levels of triglycerides, total cholesterol (TC), low-density lipoprotein cholesterol (LDL), and high-density lipoprotein cholesterol (HDL) than children of normal weight [8,9]. Friedemann et al [10] found that higher BMI was associated with adverse cardiovascular risk markers, including blood pressure, lipids, and insulin. Without effective intervention, these adverse lifestyle behaviors and early cardiometabolic risk factors may persist into adulthood and increase the future risk of cardiovascular diseases and type 2 diabetes [11].

Regular physical exercise has been repeatedly shown to be highly effective in improving cardiovascular function and metabolic markers [12-15]. However, approximately 80% of adolescents worldwide have insufficient levels of physical activity, and many adolescents spend 2 hours or more per day engaging in recreational screen time [16]. In practical situations, especially among children and adolescents who are overweight or obese, weight stigma, lack of enjoyment, and lower motor competence may reduce their willingness to participate in physical activity [17-19]. These factors may further limit sustained participation in physical activity and thereby adversely affect cardiometabolic health. These practical barriers highlight the need for interventions that can both increase physical activity and provide sufficient engagement to promote sustained participation and exercise adherence. Against this backdrop, active video games (AVG), also known as exergaming, have developed into an intervention model that integrates digital entertainment with physical activity and can significantly enhance children’s and adolescents’ interest in physical activity [20,21]. By using bodily movements to control virtual characters or advance game progress through a screen [22], these games can transform some traditionally sedentary screen-based activities into movement-based gameplay, thereby increasing energy expenditure and helping children and adolescents break sedentary habits and increase their physical activity levels [23,24].

Previous systematic reviews and meta-analyses of AVG have primarily focused on physical activity, weight management, and anthropometric outcomes. Ho et al [23], Oliveira et al [25], and Bourke et al [26] mainly evaluated physical activity levels, BMI, body weight, body fat, or other weight management-related outcomes, with limited attention to broader cardiometabolic outcomes such as blood pressure, lipid profiles, and glucose metabolism. Some reviews have begun to extend their scope to cardiometabolic outcomes, but important limitations remain. Comeras-Chueca et al [27] primarily evaluated body composition, health-related physical fitness, and motor competence in children and adolescents with overweight or obesity, whereas Erçelik et al [28] focused mainly on a limited range of outcomes, including BMI and TC, in adolescents with overweight or obesity. More recently, Hernandez-Martinez et al [29] comprehensively evaluated morphological variables, blood pressure, and biochemical parameters in children and adolescents with overweight or obesity; however, the study population was restricted to those with overweight or obesity. Zhu et al [30], in contrast, specifically evaluated blood pressure outcomes in children and young adults aged 6 to 25 years without simultaneously synthesizing other metabolic outcomes.

### Objectives

Therefore, there remains a need for a systematic review and meta-analysis based on RCTs that includes children and adolescents across a broader range of weight status and evaluates anthropometric, cardiovascular, and metabolic outcomes within a unified framework. In addition, whether intervention type and participant characteristics are associated with the effectiveness of AVG has not been consistently or sufficiently evaluated across different cardiometabolic outcomes. With the publication of additional RCTs, an updated synthesis of the available evidence is warranted to clarify the potential cardiometabolic effects of AVG and the remaining uncertainties in the current evidence base.

## Methods

### Overview

This systematic review strictly adheres to the 2020 PRISMA (Preferred Reporting Items for Systematic Reviews and Meta-Analyses) guidelines [31]. The completed PRISMA 2020 checklist is available in Appendix S1 of Multimedia Appendix 1. Additionally, this review protocol has been registered in the PROSPERO International Registry of Systematic Reviews under registration number CRD420261303390.

### Eligibility Criteria

Inclusion and exclusion criteria were established based on the population, intervention, comparison, outcomes, and study design (PICOS) framework as follows: (1) study participants were children and adolescents aged 4‐18 years; (2) participants who were able to safely engage in physical activity or interact with the intervention equipment were included, regardless of their baseline weight status or cardiometabolic health condition; (3) the intervention consisted of AVG or virtual reality (VR)-based exercise involving physical interaction with screen-based technology; (4) the control group (CG) consisted of participants who received no intervention, played sedentary video games, or maintained their regular physical activity; (5) studies reported at least 1 prespecified cardiometabolic outcome, including anthropometric measures, blood pressure, cardiorespiratory fitness, cardiac function-related measures, or metabolic biomarkers. The outcomes included in the quantitative syntheses were BMI, systolic blood pressure (SBP), diastolic blood pressure (DBP), TC, triglycerides, HDL, LDL, heart rate (HR), and glucose, as detailed in the Data Items subsection; (6) study designs were randomized controlled trials (RCTs).

Exclusion criteria included (1) studies involving adult participants outside the child and adolescent age range, and animal studies were excluded during title and abstract screening; (2) qualitative research, systematic reviews, meta-analyses, study protocols, gray literature, and abstracts only; (3) ongoing or incomplete trials without outcome data, studies lacking sufficient numerical data for effect-size calculation, and reports for which the full text could not be obtained were excluded.

### Data Items

Data were sought for all cardiometabolic outcomes prespecified in the registered protocol. The predefined outcome domains included anthropometric outcomes (BMI and body mass index z score [BMI-z]), blood pressure outcomes (SBP and DBP), cardiac and cardiorespiratory outcomes (HR, cardiorespiratory fitness, and cardiac function-related measures), and metabolic biomarkers (TC, triglycerides, HDL, LDL, glucose, insulin, and glycated hemoglobin [HbA_1c_]). The assessment conducted at the end of the intervention was used for quantitative synthesis. Outcomes were pooled when numerical data were available from at least 2 independent studies and the outcome definitions and measurement methods were sufficiently comparable. On this basis, the quantitative syntheses included BMI, SBP, DBP, TC, triglycerides, HDL, LDL, HR, and glucose.

Other extracted variables included study author and publication year, participant sample size, age, sex distribution, intervention and comparator characteristics, intervention frequency, session duration, total intervention duration. When information was missing or insufficiently reported, it was recorded as not reported unless it could be derived directly from the published numerical data using standard statistical conversions. Unreported values were not imputed.

### Information Sources

The databases searched were PubMed, Web of Science, the Cochrane Library, and Embase. Eligible studies were required to be peer-reviewed, published in English, accessible in full text, and without restrictions on publication date or sample size. Searches were conducted in July 2026.

### Search Strategy

The search strategy was developed and reported in accordance with the PRISMA-S (Preferred Reporting Items for Systematic Reviews and Meta-Analyses literature search) extension for reporting literature searches in systematic reviews [32]. The search strategy was developed based on 3 key concepts: children and adolescents, AVG, and cardiometabolic outcomes. Controlled vocabulary terms were used, including MeSH in PubMed and Emtree terms in Embase, combined with free-text terms. The full search strategies are provided in Supplementary Material S2 of Multimedia Appendix 1. Additional searches included backward and forward citation searching of included studies. PROSPERO and the Cochrane Library were also searched to identify relevant registered or published systematic reviews.

### Selection Process

The retrieved literature was first manually deduplicated by an independent researcher (LWQ) using EndNote 21 (Clarivate). After deduplication, 2 independent researchers (LWQ and WYX) screened the titles and abstracts against the predefined inclusion and exclusion criteria. In case of disagreement, the 2 researchers discussed the matter; if consensus could not be reached, a third independent researcher (WTL) made the final decision. Subsequently, the 2 researchers reviewed the full texts of the potentially eligible studies to determine the final included studies. Any disagreements arising at this stage were resolved using the same discussion and arbitration process. Other potentially eligible studies included those mentioned in previous relevant systematic reviews but not initially identified, or studies known to the research team through professional experience that met the inclusion criteria.

### Data Collection Process

Data were independently extracted by 2 researchers (LWQ and WYX) using a predesigned Excel (Microsoft) data extraction form. Any disagreements were reviewed by a third researcher (WTL), and unresolved disagreements were adjudicated by a fourth independent researcher (LJ). If data were missing or presented only in graphical form, authors were contacted to obtain raw data; if contact was unsuccessful, data were extracted from the figures using Web Plot Digitizer 4.1 (Automeris). The reliability and validity of this software have been previously demonstrated [33]. Studies without sufficient data for effect size calculation were excluded. Extracted data included the mean, SD, and sample size for each group before and after the intervention. During data synthesis, when multiple eligible intervention groups shared a common CG, the eligible intervention groups were combined where appropriate to avoid double counting of the control participants, in accordance with the Cochrane Handbook [34].

When weight status classifications were not explicitly reported, BMI or BMI-z scores were used to determine whether participants met the criteria for overweight or obesity. BMI-z scores were not pooled with BMI values in the main meta-analysis. Although BMI is expressed in kg/m², its clinical interpretation varies according to age, sex, and developmental stage in children and adolescents. Therefore, BMI outcomes were analyzed using Hedges *g* to standardize effects across studies, and the pooled BMI effect was interpreted as a standardized difference rather than a direct change in BMI values.

### Study Risk of Bias Assessment

The Cochrane Risk of Bias 2 tool (RoB 2) was used to assess the risk of bias in RCTs [35], examining the following domains: bias arising from the randomization process; bias due to deviations from intended interventions; bias due to missing outcome data; bias in measurement of the outcome; and bias in selection of the reported result. RoB 2 judgments were categorized as “Low risk of bias,” “Some concerns,” or “High risk of bias” in accordance with the official RoB 2 terminology. Any disagreements were resolved through discussion and, if necessary, by third-party arbitration.

Additionally, the Physiotherapy Evidence Database (PEDro) scale was used to assess methodological quality [36], with a scoring range of 0‐10 points: ≥6 points indicated high quality, 4‐5 points indicated moderate quality, and ≤3 points indicated low-quality studies. PEDro scores were used only as supplementary descriptive information on methodological quality and were not used as the basis for GRADE (Grading of Recommendations Assessment, Development and Evaluation) downgrading.

### Sensitivity Analyses

Sensitivity analyses were conducted to assess the robustness of the pooled estimates, following established guidance for meta-analytic sensitivity and influence analyses [34]. Using a leave-one-out approach [37], each study was sequentially excluded, and the restricted maximum likelihood (REML)–based random-effects model with the Hartung-Knapp adjustment [38] was re-estimated. For each iteration, the pooled Hedges *g*, 95% CI, *I*^2^, and τ^2^ were recalculated. Robustness was assessed by examining whether omission of any single study materially altered the direction, magnitude, precision, heterogeneity, or overall interpretation of the pooled effect. Changes in statistical significance were considered but were not used as the sole criterion for judging robustness.

### Synthesis Methods

A random-effects model was applied because clinical and methodological heterogeneity among studies was anticipated, consistent with current recommendations for rigorous meta-analysis methodology [39]. Between-study variance (τ²) was estimated using the REML method, 95% CI for the pooled effects were calculated using the Hartung-Knapp-Sidik-Jonkman adjustment [38], and 95% prediction intervals (PIs) were obtained using the Nagashima-Noma-Furukawa method [40]. Since the outcome measures in this study involved various units, and previous studies had recommended prioritizing standardized effect sizes, standardized mean differences (Hedges *g*) were used. Given that most included studies have small sample sizes, Hedges *g* was calculated using a bias-corrected exact formula as the effect size measure. Hedges *g* is interpreted as follows: 0.2 indicates a small effect, 0.5 indicates a moderate effect, and 0.8 indicates a large effect. Statistical heterogeneity was characterized using Cochran’s Q, *τ*², and 95% PI, while *I*² was used as a relative measure of inconsistency among study estimates. *I*² was interpreted in relation to sampling error rather than as an absolute measure of between-study heterogeneity. Accordingly, fixed thresholds were not used to categorize *I*² values; instead, *I*² was interpreted descriptively in conjunction with τ², the *P* value from Cochran’s *Q* test, and PI [41]. Outcomes were quantitatively synthesized when numerical data were available from at least 2 independent studies and the outcome definitions and measurement methods were sufficiently comparable [34]. Forest plots were used to display the individual study estimates and pooled effects, including for outcomes supported by few studies. These results were interpreted cautiously because of the limited precision of the available evidence.

Prespecified subgroup analyses were considered only when at least 10 studies were available for an outcome and were regarded as exploratory [34]. Accordingly, subgroup analyses were conducted only for BMI. No formal subgroup inference was made when a subgroup contained only one study.

Small-study effects were examined using funnel plots and Egger’s regression test only when at least 10 studies were available for an outcome [42]. Accordingly, these analyses were performed only for BMI. A nonsignificant test result was interpreted as indicating no statistical evidence of small-study effects, rather than evidence that small-study effects were absent. Subgroup analyses and small-study effects tests were interpreted cautiously when only a limited number of studies or effect sizes were available, to reduce the risk of low statistical power and unstable inferences due to an insufficient number of studies. Statistical analysis and visualization were performed using the “meta” and “metafor” packages in R software (R Foundation for Statistical Computing) (version 4.2.0). Statistical significance was set at *P*<.05.

### Certainty Assessment

The certainty of evidence for each outcome was assessed using the GRADE approach [43]. The GRADE approach classifies the certainty of evidence as high, moderate, low, or very low. Evidence certainty was rated based on 5 domains: risk of bias, inconsistency, indirectness, imprecision, and publication bias. The initial assessment was conducted by 1 reviewer (LWQ) and independently cross-checked by a second reviewer (WYX).

## Results

### Study Selection

The database search identified a total of 2307 records. Among these, 2301 were imported from PubMed, Web of Science, Cochrane Library, and Embase, while the remaining 6 records were identified through forward and backward citation searching within the final literature. After removing duplicate studies and screening titles or abstracts, 72 full-text articles were assessed for eligibility, and ultimately 14 RCTs were included in this systematic review and meta-analysis [44-57]. The study selection process is shown Figure 1.

**Figure 1.**
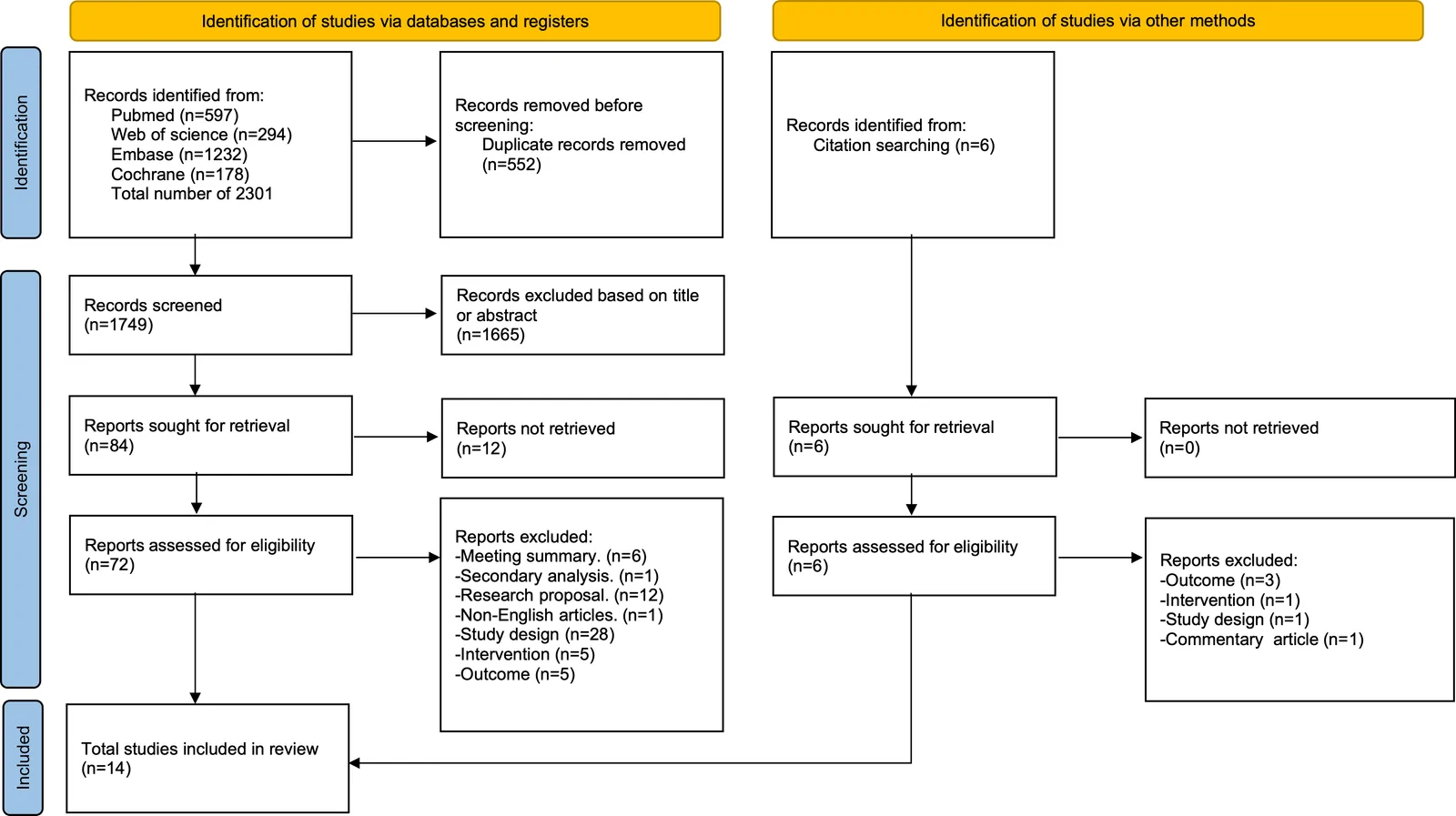
PRISMA (Preferred Reporting Items for Systematic Reviews and Meta-Analyses) flowchart for the search strategy.

### Study Characteristics

A total of 14 RCTs were included, with publication years ranging from 2009 to 2026. The included studies were conducted in Turkey (n=1) [47], New Zealand (n=1) [52], China (n=2) [50,57], Spain (n=1) [48], the United States (n=6) [46,49,51,54-56], Tunisia (n=1) [44], Norway (n=1) [45], and Italy (n=1) [53]. The total sample size was 942 participants, with individual study sample sizes ranging from 22 to 231 participants. Five studies [45,46,52,55,56] reported the sex distribution at baseline but did not report the sex distribution of the final analyzed sample. The mean age of participants ranged from 4 to 16 years. Participants included children and adolescents with normal weight, overweight, or obesity; one study included participants with endothelial dysfunction (EDF) [54]. BMI values ranging from 15 to 38 kg/m² across studies.

Interventions primarily included AVG and VR exercise, the CG mostly engaged in usual physical activity or received no intervention. Training frequency was typically 3‐5 times per week, with intervention durations ranging from 4‐24 weeks. Outcome measures included BMI, HR, SBP, DBP, HDL, LDL, TC, triglycerides, and glucose (Table 1).

**Table 1.** Characteristics of the included studies.

Author, year	Sample			Group	Participant age^b^ (years)	Intervention	Comparator	Outcome	Frequency (times/week)	Time (each time)	Duration (week)
	Total^a^	Female	Male								
Coknaz et al, 2019 [47]	106	60	46	AVG^c^; CG^d^	AVG: 9.62 (1.02); CG: 10.31 (1.15)	AVG	Did not play games	BMI	3	50‐60	12
Maddison et al, 2011 [52]	231	87^t^	235	AVG; CG	AVG: 11.6 (1.1); CG: 11.6 (1.1)	AVG	Video game	BMI	NR^e^	60	24
Wang et al, 2025 [57]	138	52	86	REVERIE; CG	AVG: 13.98 (1.77); CG: 14.17 (1.85)	VR^f^	Usual PA^g^	BMI, HR^h^, SBP^i^, DBP^j^, TC^k^, triglycerides, HDL^l^, LDL^m^	3	45	8
Comeras−Chueca et al, 2022 [48]	28	13	15	AVG; CG	AVG: 10.2 (0.8); CG: 9.7 (0.8)	AVG and multicomponent training	Usual PA	BMI	3	60	20
Murphy et al^n^, 2009 [54]	35	17	18	EX^o^; DTC^p^	AVG: 9.23 (1.56); CG: 9.23 (1.56)	Dance dance revolution	Usual PA	BMI, SBP, DBP, TC, triglycerides, LDL, HDL, glucose	5	10‐30	12
Abedelmalek et al, 2022 [44]	24	0	24	EG^q^; CG	AVG: 15.75 (0.8); CG: 16.25 (0.8)	Cooperative exergame	Without additional exercise	BMI, TC, triglycerides, HDL, LDL	5	45	4
Christison et al, 2016 [46]	48	46^t^	34	Exergaming group; CG	AVG: 10.1 (1.3); CG: 10.0 (1.2)	Exergaming and traditional exercise	Received didactic curriculum only	BMI, HR, SBP	5	60	24
Gao et al, 2019 [49]	32	16	16	Exergaming; CG	AVG: 4.56 (0.62); CG: 4.93 (0.83)	Exergaming	Usual PA	BMI	5	30	12
Staiano et al, 2018 [55]	45	21^t^	25	Intervention; CG	AVG: 11.2 (0.8); CG: 11.2 (0.8)	Exergaming	Usual PA	Triglycerides, HDL, LDL, glucose	3	60	24
Staiano et al, 2017 [56]	38	41^t^^r^	0	Intervention; CG	AVG: 15.3 (1.2); CG: 16.1 (1.4)	Dance exergaming	Usual PA	Triglycerides, HDL, LDL, glucose	3	60	12
Lau et al, 2016 [50]	80	25	55	AVG; CG	AVG: 9.23 (0.52); CG: 9.23 (0.52)	School-based active video game	Usual PA	BMI	2	60	12
Berg et al, 2026 [45]	22	2^t^	27	Exergaming; CG	AVG: 13.0 (1.3); CG: 14.3 (2.0)	Cycling exergaming	No intervention	BMI, SBP, DBP, TC, triglycerides, HDL, LDL, glucose	With no mandatory sessions	With no mandatory sessions	24
Lu et al, 2025 [51]	51	21	30	AVG; Waitlist control	AVG: 10.94 (1.73); CG: 10.59 (1.70)	Home-based AVG	Usual PA	HDL, LDL, triglycerides, glucose	1‐7	30‐60	24
Marsigliante et al, 2024 [53]	64	0	64	EG; CG	AVG: 9.5 (0.6); CG: 9.5 (0.8)	Exergames	Usual PA	BMI	3	45	24

a Sample size included in the final analysis.

b All data are presented as mean (SD).

c AVG: active video games.

d CG: control group.

e NR: not reported.

f VR: virtual reality.

g PA: physical activity.

h HR: heart rate.

i SBP: systolic blood pressure.

j DBP: diastolic blood pressure.

k TC: total cholesterol.

l HDL: high-density lipoprotein.

m LDL: low-density lipoprotein.

n The study subjects have endothelial dysfunction (EDF).

o EX: exercise group.

p DTC: delayed treatment CG.

q EG: exergaming.

r Sex distribution reported at baseline.

### Results of Individual Studies

In the pooled analysis of 14 studies [44-57], AVG were associated with a small but statistically significant reduction in BMI (n=899; Hedges *g*=−0.23, 95% CI −0.35 to −0.10; *P*=.002). The PI (95% PI −0.43 to −0.01) remained entirely below the null, indicating that the direction of the estimated effect was consistent across the range of plausible true effects (Figure 2). SBP (n=243; Hedges *g*=−0.18, 95% CI −0.36 to −0.001; *P*=.0497) showed a small statistically significant reduction; however, the PI (95% PI −0.43 to 0.07) crossed the null, indicating uncertainty regarding whether a similar effect would be observed across different future settings (Figure 3). The pooled estimate did not show a significant reduction in TC (n=208; Hedges *g*=−0.96, 95% CI −3.79 to 1.88; *P*=.37), the estimate was highly uncertain, with *I*^2^=87% (Figure 4).

No statistically significant effects were observed for HR (n=186; Hedges *g*=−0.14, 95% CI −1.74 to 1.46; *P*=.47), DBP (n=195; Hedges *g*=−0.11, 95% CI −0.86 to 0.63; *P*=.59), TG (n=342; Hedges *g*=−0.20, 95% CI −0.70 to 0.31; *P*=.38), HDL (n=342; Hedges *g*=0.05, 95% CI −0.21 to 0.31; *P*=.65), LDL (n=342; Hedges *g*=−0.26, 95% CI −0.64 to 0.13; *P*=.15), or glucose (n=180; Hedges *g*=−0.17, 95% CI −0.37 to 0.02; *P*=.07). See Figures 5–10.

*I*^2^ values ranged from 0% to 87% across outcome measures, while PI provided additional information regarding the range of plausible effects in future similar settings. The highest *I*^2^ value was observed for TC (*I*²=87%), whereas *I*^2^ was 0% for BMI, HR, SBP, HDL, and glucose.

**Figure 2.**
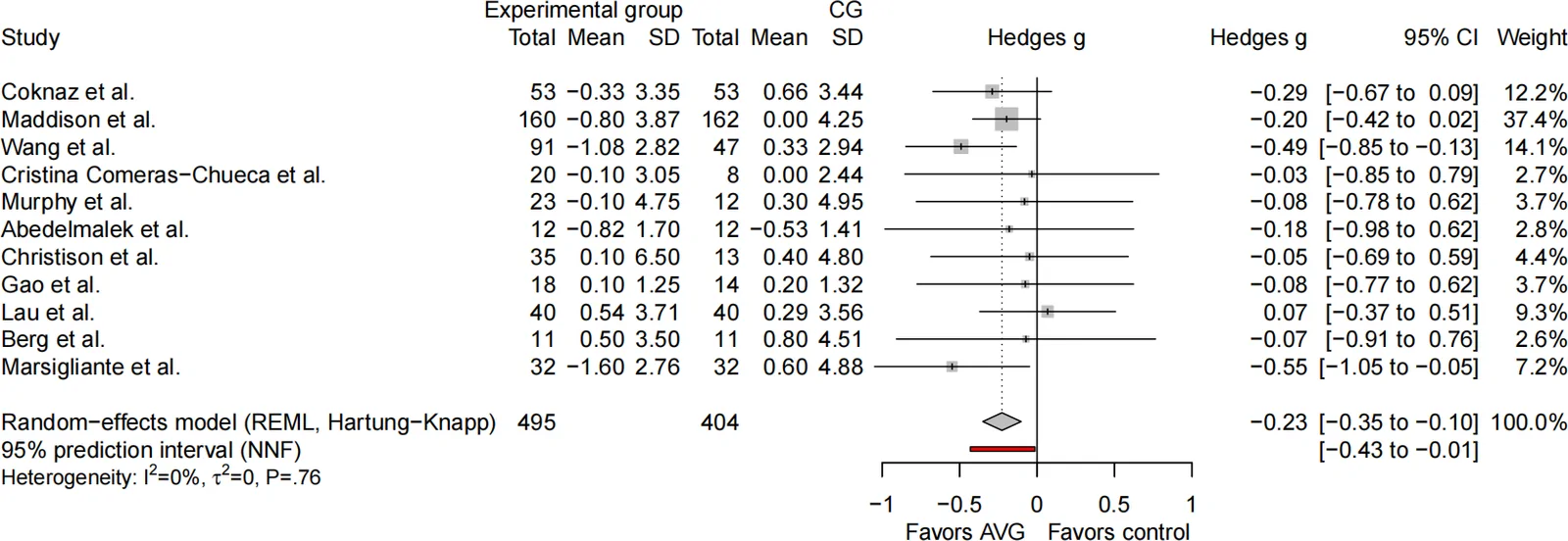
Forest plot of the effects of AVG on BMI in children and adolescents [44-50,52-54,57]. Hedges *g*: standardized mean difference corrected for small-sample bias*. I*^2^: relative measure of inconsistency; *τ*²: between-study variance; AVG: active video games; CG: control group; NNF: Nagashima-Noma-Furukawa; REML: random-effects model.

**Figure 3.**
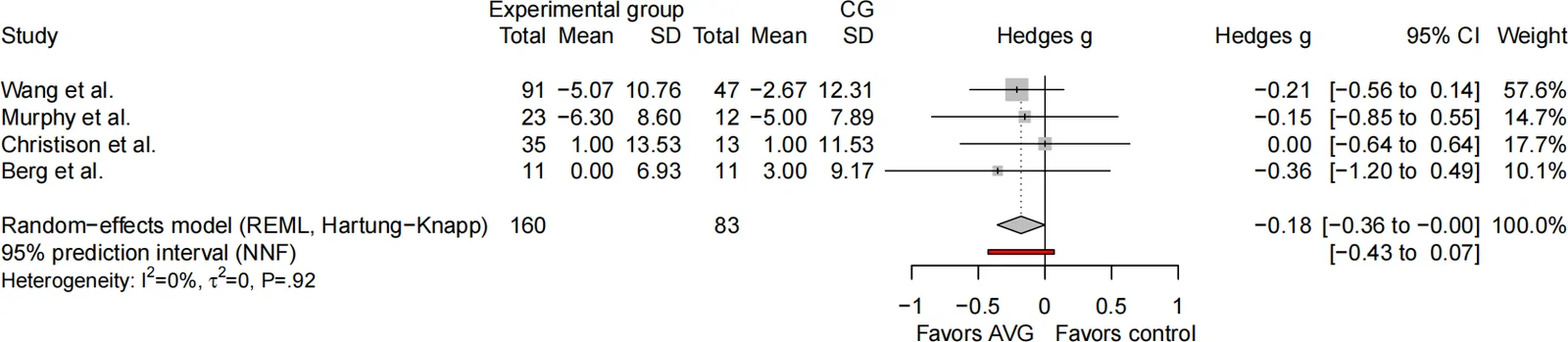
Forest plot of the effects of AVG on SBP in children and adolescents. Hedges *g*: standardized mean difference corrected for small-sample bias*.* Estimates in the forest plot are presented to 2 decimal places. For SBP, the upper 95% CI limit (−0.001) and *P* value (*P*=.0497) are reported with additional precision in the main text because rounding would yield −0.00 and *P*=.05, which could obscure the inferential result [45,46,54,57]. *I*^2^: relative measure of inconsistency; τ²: between-study variance; AVG: active video games; CG: control group; NNF: Nagashima–Noma–Furukawa; REML: random-effects model; SBP: systolic blood pressure.

**Figure 4.**
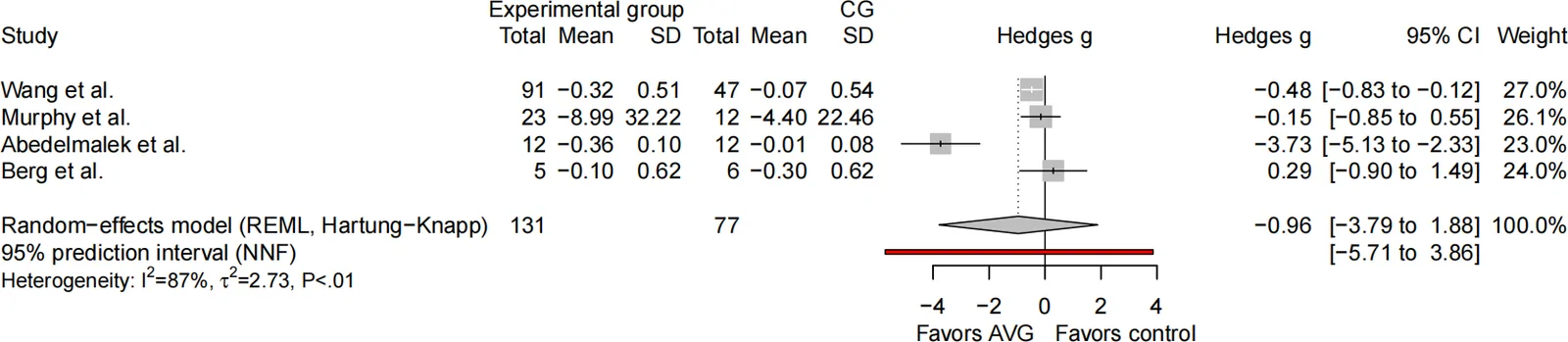
Forest plot of the effects of AVG on TC in children and adolescents [44,45,54,57]. Hedges *g*: standardized mean difference corrected for small-sample bias. *I*^2^: relative measure of inconsistency; τ²: between-study variance; AVG: active video games; CG: control group; NNF: Nagashima–Noma–Furukawa; REML: random-effects model; TC: total cholesterol.

**Figure 5.**
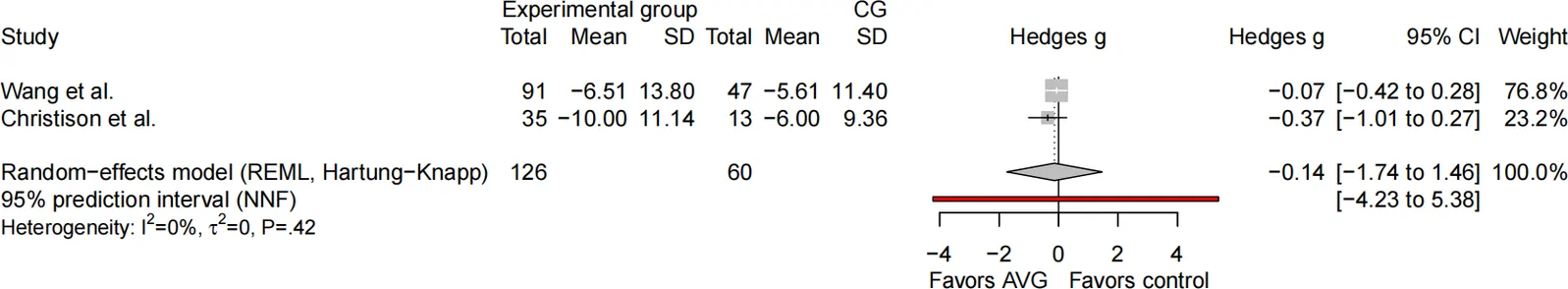
Forest plot of the effects of AVG on HR in children and adolescents [46,57]. Hedges *g*: standardized mean difference corrected for small-sample bias []. *I*^2^: relative measure of inconsistency; τ²: between-study variance; AVG: active video games; CG: control group; HR: heart rate; NNF: Nagashima–Noma–Furukawa; REML: random-effects model.

**Figure 6.**
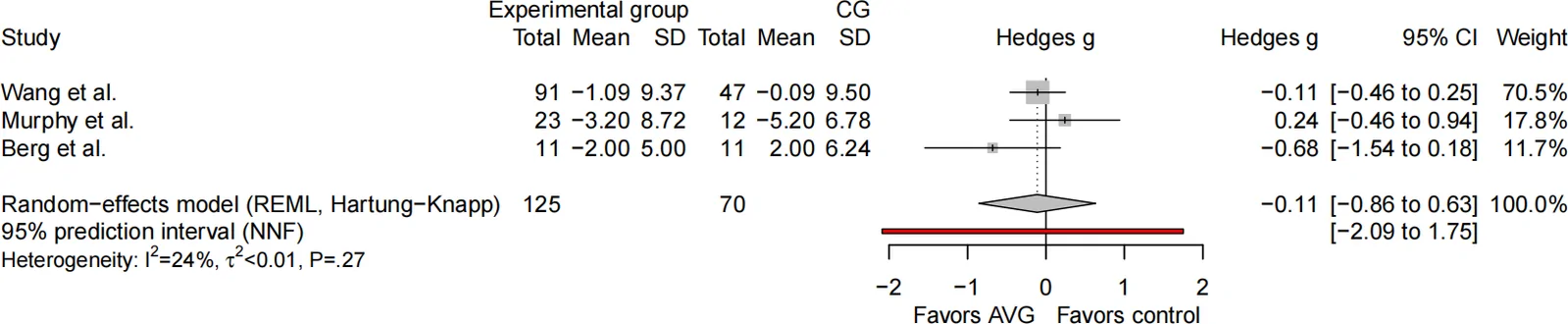
Forest plot of the effects of AVG on DBP in children and adolescents [45,54,57]. Hedges *g*: standardized mean difference corrected for small-sample bias; *I*^2^: relative measure of inconsistency; τ²: between-study variance; AVG: active video games; CG: control group; DBP: diastolic blood pressure; NNF: Nagashima-Noma-Furukawa; REML: random-effects model.

**Figure 7.**
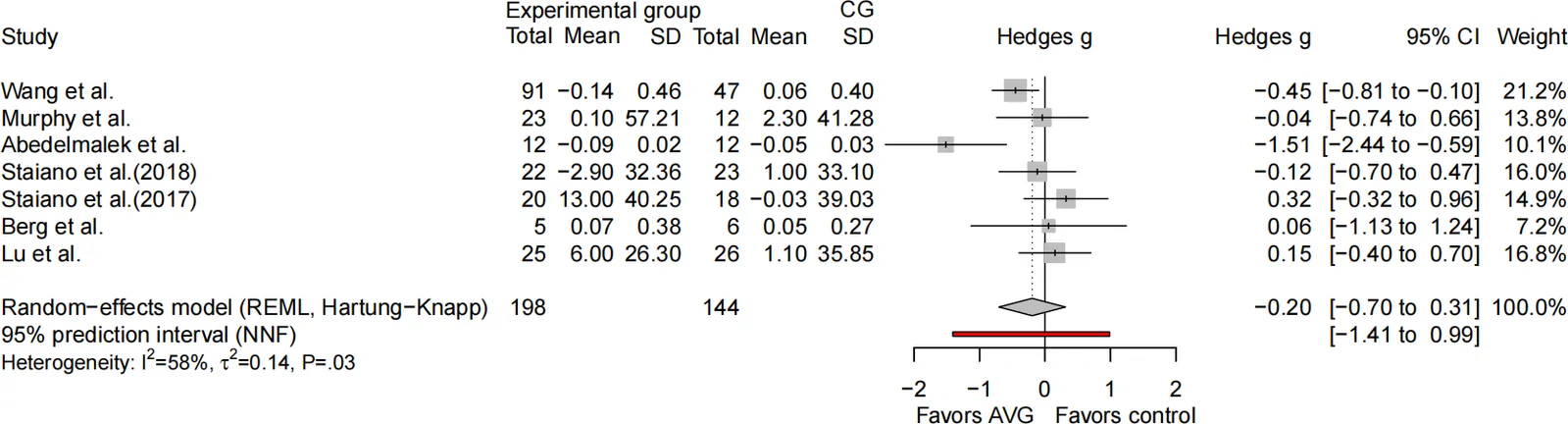
Forest plot of the effects of AVG on triglycerides in children and adolescents [44,45,51,54-57. Hedges *g*: standardized mean difference corrected for small-sample bias. *I*^2^: relative measure of inconsistency; τ²: between-study variance; AVG: active video games; CG: control group; NNF: Nagashima-Noma-Furukawa; REML: random-effects model.

**Figure 8.**
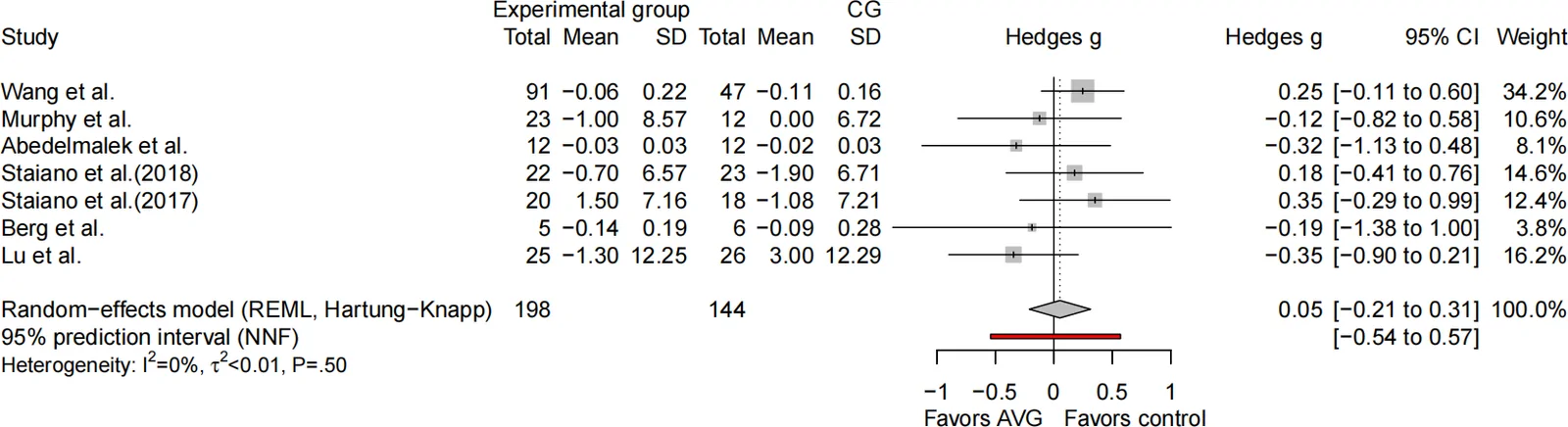
Forest plot of the effects of AVG on HDL in children and adolescents [44,45,51,54-57]. Hedges *g*: standardized mean difference corrected for small-sample bias. *I*^2^: relative measure of inconsistency; τ²: between-study variance; AVG: active video games; CG: control group; HDL: high-density lipoprotein cholesterol; NNF: Nagashima-Noma-Furukawa; REML: random-effects model.

**Figure 9.**
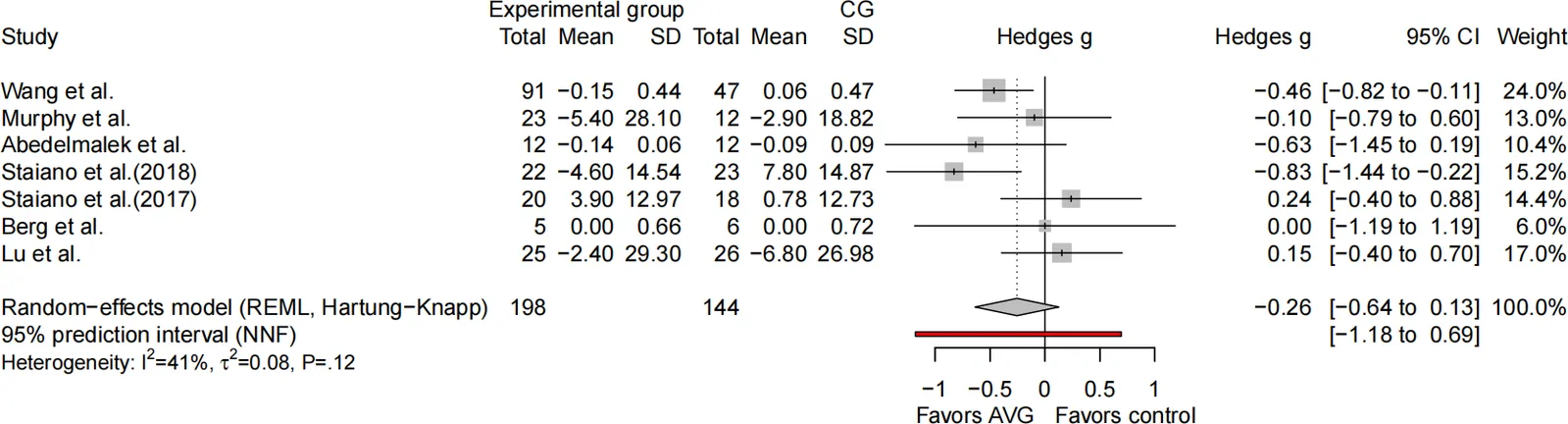
Forest plot of the effects of AVG on LDL in children and adolescents [44,45,51,54-57]. Hedges *g*: standardized mean difference corrected for small-sample bias; *I*^2^: relative measure of inconsistency; τ²: between-study variance; AVG: active video games; CG: control group; LDL: low-density lipoprotein cholesterol; NNF: Nagashima-Noma-Furukawa; REML: random-effects model.

**Figure 10.**
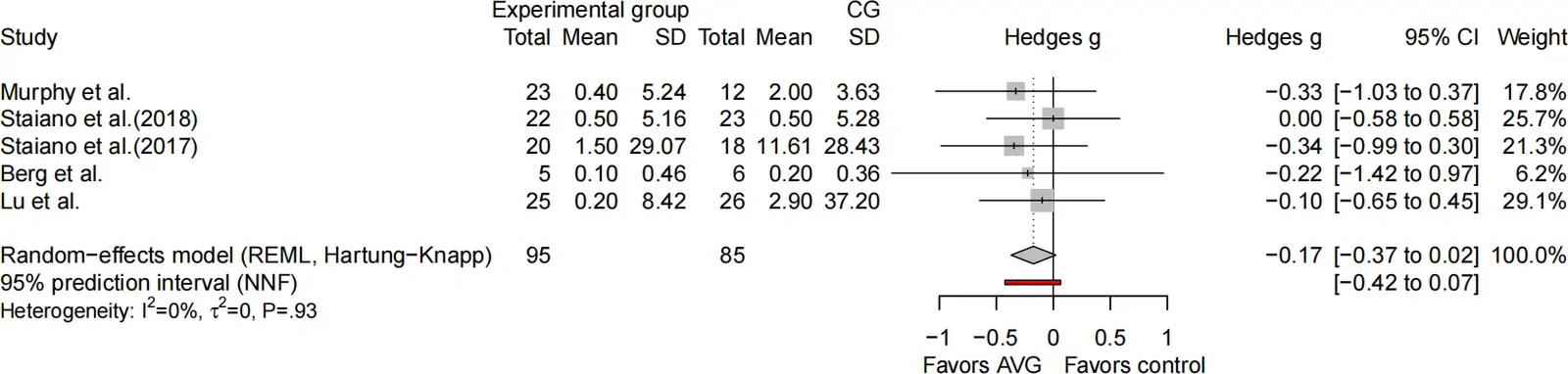
Forest plot of the effects of AVG on glucose in children and adolescents [45,51,54-56]. Hedges *g*: standardized mean difference corrected for small-sample bias; *I*^2^: relative measure of inconsistency; τ²: between-study variance; AVG: active video games; CG: control group; NNF: Nagashima-Noma-Furukawa; REML: random-effects model.

### Subgroup Analysis

To further explore potential sources of heterogeneity and examine whether participant characteristics and intervention-related factors may modify the effects of AVG on cardiometabolic health in children and adolescents, subgroup analyses were conducted for BMI according to 3 dimensions: sex, intervention type, and nutritional status. These findings should be interpreted cautiously due to the limited number of studies within some subgroups.

Subgroup analysis revealed a small but statistically significant effect favoring BMI reduction in the mixed-sex group (Hedges *g*=−0.20), whereas it was not statistically significant in the male group (Hedges *g*=−0.45), likely reflecting the limited number of included studies (*K*=2) and the imprecision of the estimate (95% CI −2.55 to 1.66). The subgroup differences by sex were not statistically significant (*P*=.17). See Figure 11. Both the AVG group (Hedges *g*=−0.18) and the VR group (Hedges *g*=−0.49) showed effects favoring BMI reduction. See Figure 12. Because the VR category comprised a single study, this estimate should be regarded as a single-study observation, and no formal inference regarding differences between AVG and VR interventions can be made. Regarding nutritional status, the pooled effects were similar across normal weight (Hedges *g*=−0.24), overweight (Hedges *g*=−0.22), and obesity subgroups (Hedges *g*=−0.22), with no significant subgroup differences (*P*=.99). See Figure 13. Overall, no statistically significant subgroup differences were observed according to sex, intervention type, or nutritional status.

**Figure 11.**
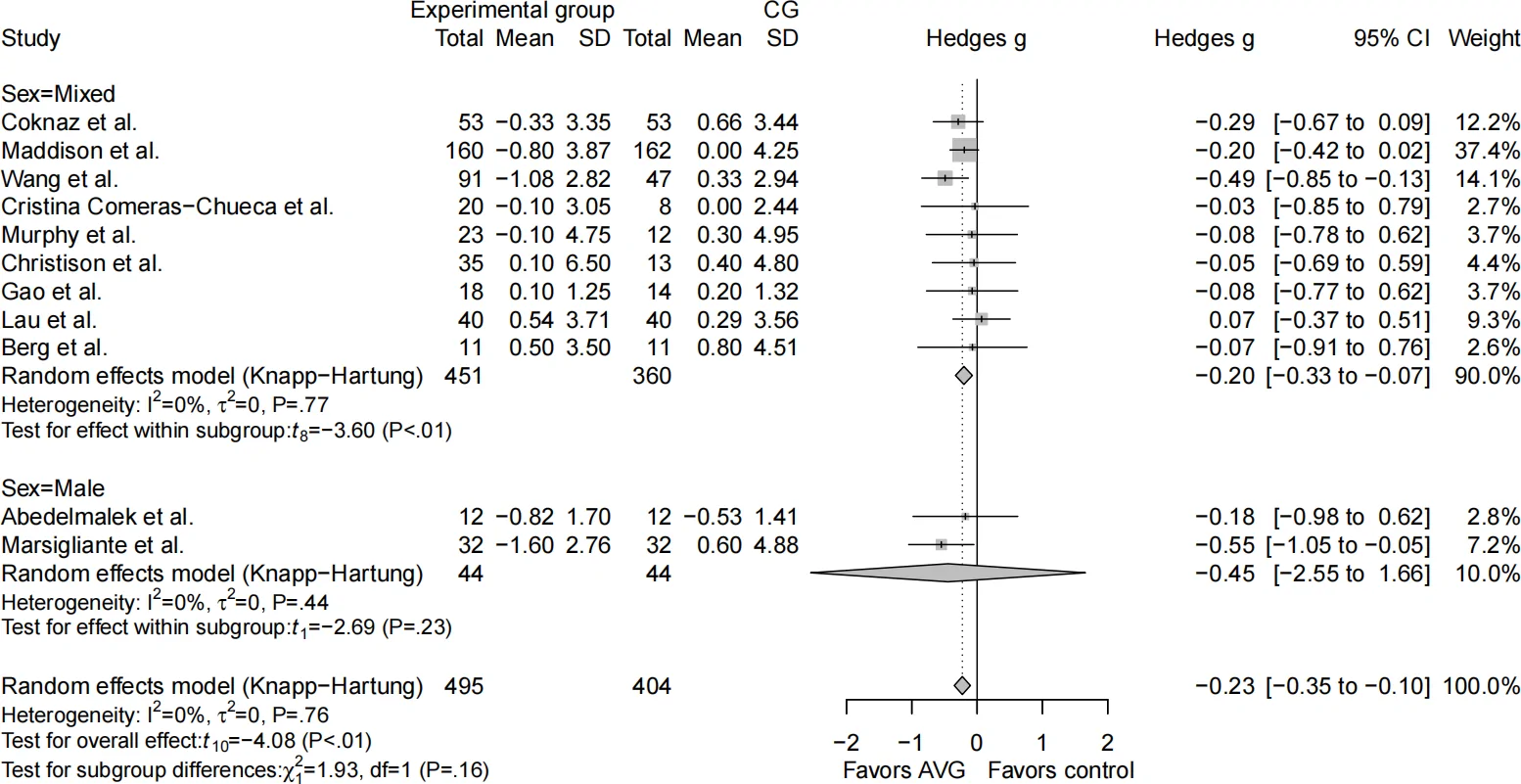
Subgroup analysis of the effects of AVG on BMI according to sex [44-50,52-54,57]. Hedges *g*: standardized mean difference corrected for small-sample bias; *I*^2^: relative measure of inconsistency; τ²: between-study variance; AVG: active video games; CG: control group; VR: virtual reality.

**Figure 12.**
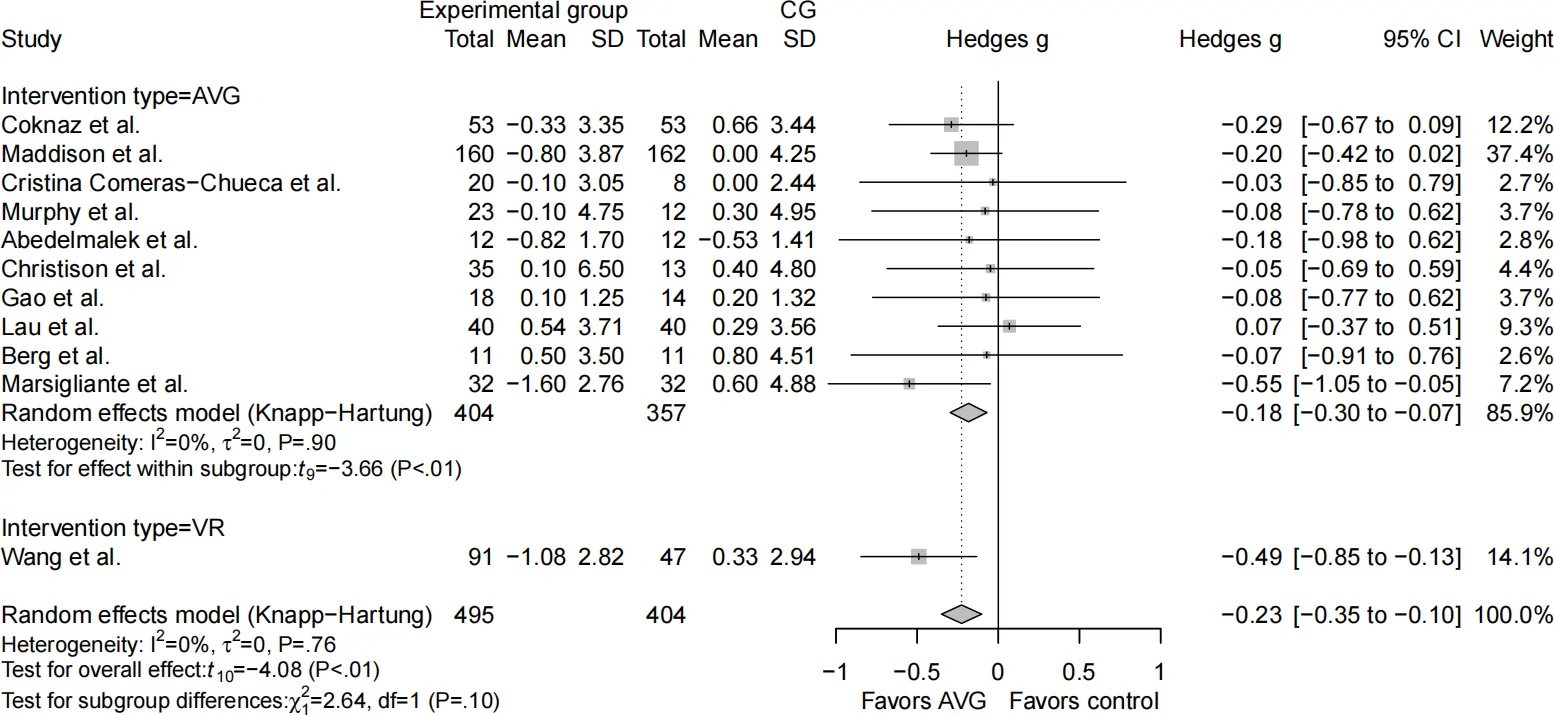
Subgroup analysis of the effects of AVG on BMI according to intervention type [44-50,52,54,57]. Hedges *g*: standardized mean difference corrected for small-sample bias; *I*^2^: relative measure of inconsistency; τ²: between-study variance; AVG: active video games; CG: control group; VR: virtual reality.

**Figure 13.**
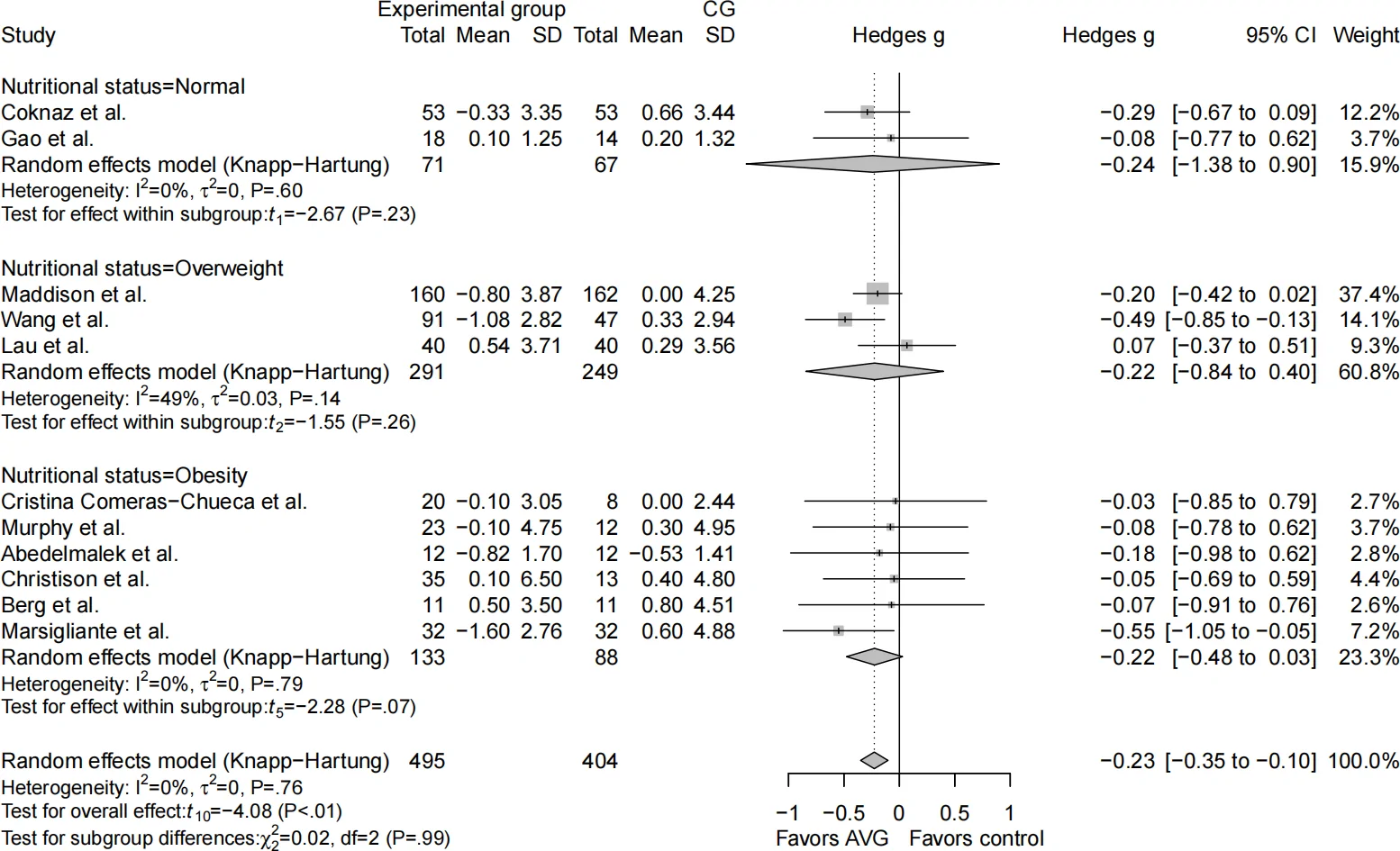
Subgroup analysis of the effects of AVG on BMI according to nutritional status [44-50,52-54,57]. Hedges *g*: standardized mean difference corrected for small-sample bias; *I*^2^: relative measure of inconsistency; τ²: between-study variance; AVG: active video games; CG: control group.

### Risk of Bias in Studies

Overall, none of the included studies were judged to have a low risk of bias; 9 studies [44,45,48-50,53-56] were classified as having some concerns, and 5 studies [45,46,52,55,57] were judged to have a high risk of bias. Bias in measurement of the outcome (D4) was consistently rated as low risk of bias, with all studies judged as low risk of bias in the domain. Bias due to deviations from intended interventions (D2) was generally rated as low risk of bias, with only Christison et al [46] being judged as having a high risk of bias. This judgment was mainly due to potential deviations from the intended intervention, including additional physical activity opportunities beyond the prescribed exergaming sessions and limited control over intervention exposure. Bias in selection of the reported result (D5) was predominantly rated as some concerns, mainly due to insufficient information regarding trial registration or prespecified analysis plans, though no study was judged high risk of bias in this domain. Bias arising from the randomization process (D1) was rated as low risk of bias in 5 studies, with 8 studies having some concerns and 1 study judged as having high risk of bias, generally reflecting incomplete reporting of allocation concealment. Among the assessed domains, missing outcome data (D3) represented the main source of high risk of bias, which was judged high risk of bias in 4 studies, mainly due to substantial missing outcome data or insufficient reporting of participant attrition during follow-up. Among the 5 studies judged to have a high overall risk of bias, four were primarily driven by concerns in D3, suggesting that missing outcome data was an important contributor to overall risk judgments. See Figure 14.

A funnel plot combined with Egger’s regression test was used to assess small-study effects for the BMI outcome (Appendix S3 in Multimedia Appendix 1). Due to the limited number of studies available for the other outcomes, small-study effects were only examined for BMI. Egger’s regression test showed no evidence of small-study effects for BMI (*P*=.46).

The average PEDro score across all studies was 5.4, indicating that the methodological quality of the included studies was generally moderate (Appendix S6 in Multimedia Appendix 1).

**Figure 14.**
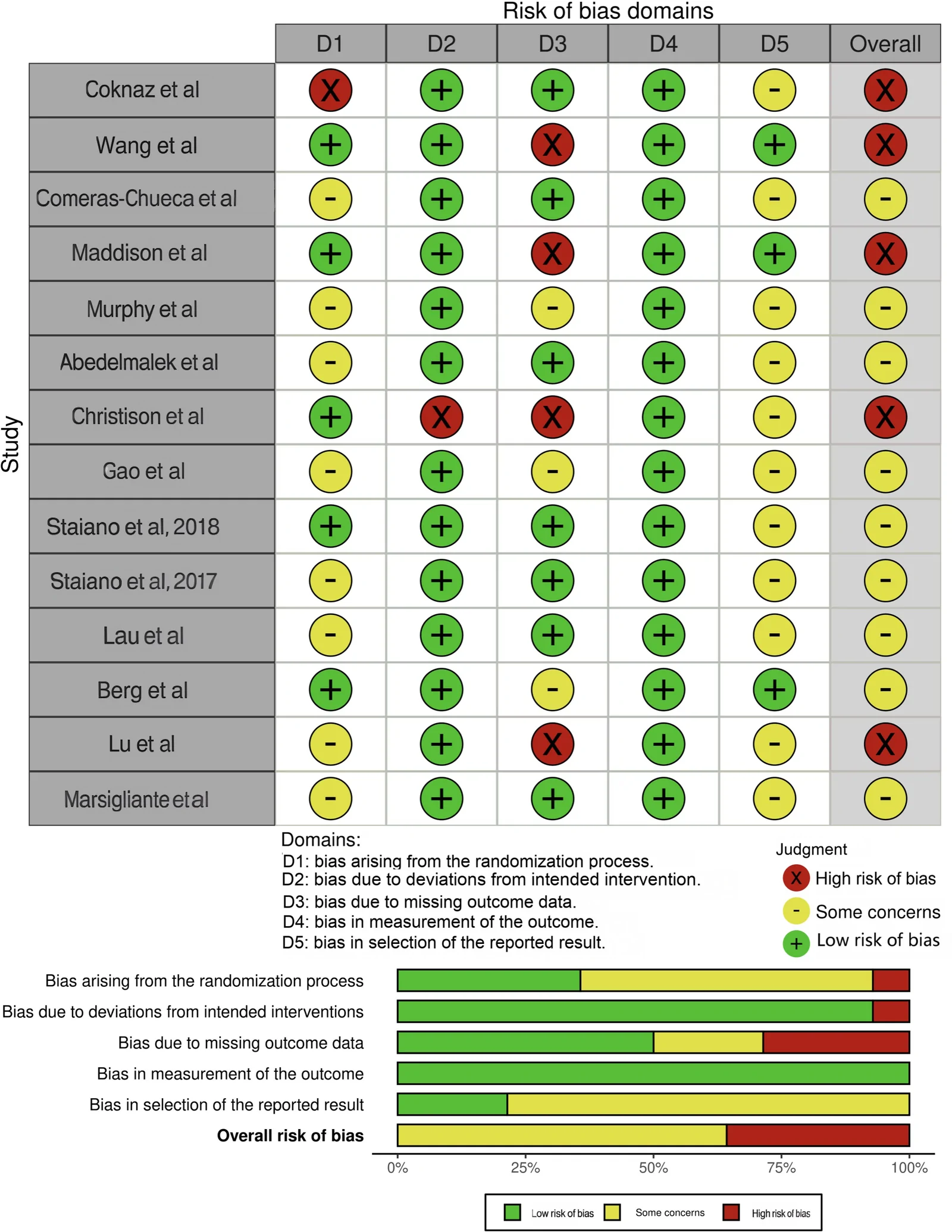
Summary of the risk of bias [44-57].

### Sensitivity Analysis

Sensitivity analyses were conducted using the leave-one-out method to examine the stability of the pooled estimates. For BMI, the overall effect was significant (Hedges *g*=−0.23, 95% CI −0.35 to −0.10; *P*=.002; *I*^2^=0%), the direction and significance of the results remained unchanged after excluding any single study, indicating that BMI demonstrated the highest robustness across all outcomes. The pooled effect for SBP showed a small statistically significant reduction (Hedges *g*=−0.18, 95% CI −0.36 to −0.001; *P*=.0497). After excluding the study by Christison et al [46], the reduction became statistically stronger, suggesting that the magnitude of the SBP effect was influenced by this study. One possible explanation may be the substantial loss to follow-up reported in this study, which could have influenced the pooled estimate.

The pooled effects for HR (Hedges *g*=−0.14, 95% CI −1.74 to 1.46; *P*=.47) and HDL (Hedges *g*=0.05, 95% CI −0.21 to 0.31; *P*=.65) were all nonsignificant, and given the limited number of included studies, the evidence was limited due to the small number of available studies. Similarly, the effects for DBP (Hedges *g*=−0.11, 95% CI −0.86 to 0.63; *P*=.59), LDL (Hedges *g*=−0.26, 95% CI −0.64 to 0.13; *P*=.15), TC (Hedges *g*=−0.96, 95% CI −3.79 to 1.88; *P*=.37), and TG (Hedges *g*=−0.20, 95% CI −0.70 to 0.31; *P*=.38) remained nonsignificant after removal of individual studies. *I*^2^ values ranged from 24% to 87% across these outcomes. For TC, *I*^2^ decreased from 87% to 0% after excluding Abedelmalek et al [44], suggesting that this study contributed to the observed inconsistency among study estimates; however, the magnitude of the pooled effect was reduced. The pooled effect for glucose (Hedges *g*=−0.17, 95% CI −0.37 to 0.02; *P*=.07) remained nonsignificant after excluding any single study.

Overall, the results of the sensitivity analysis suggest that the improvement in BMI is robust. The evidence regarding blood pressure, glucose, and other lipid markers is insufficient. Future studies of higher quality are needed to validate these findings. Refer to Appendix S4 in Multimedia Appendix 1 for details.

### Certainty of Evidence

The quality of evidence was assessed using the GRADE framework. Detailed results are presented in Supplementary Material S5 of Multimedia Appendix 1. Overall, the quality of evidence ranged from low to very low primarily due to factors such as risk of bias, inconsistency, and imprecision. Specifically, the certainty of evidence for BMI and SBP was rated as low, primarily due to serious risk of bias, whereas no serious concerns were identified regarding inconsistency, indirectness, or imprecision. The certainty of evidence for DBP was also rated as low, downgraded mainly because of concerns regarding risk of bias and imprecision. For HR, HDL, LDL, and glucose, the certainty of evidence was mainly downgraded due to serious risk of bias and imprecision. For TC and TG, additional downgrading was applied due to inconsistency and imprecision. For several cardiometabolic outcomes, the CI included the null value, indicating substantial uncertainty in the estimated effects. Risk-of-bias downgrading was based on RoB 2 judgments, whereas PEDro scores were retained only for descriptive methodological context.

## Discussion

### Principal Findings

This systematic review and meta-analysis aimed to evaluate the effects of AVG on cardiometabolic health in children and adolescents, and to explore potential moderators through subgroup analysis. The main findings indicated that AVG were associated with a small but statistically significant reduction in BMI. Subgroup analyses suggested that sex, intervention type, and nutritional status did not significantly modify the effect of AVG on BMI. Sensitivity analyses supported the robustness of the pooled BMI estimate. The certainty of evidence was rated as low due to concerns regarding risk of bias.

AVG were associated with a small reduction in SBP. No statistically significant effects were observed for HDL, LDL, TC, triglycerides, DBP, HR, and glucose. The certainty of evidence for most cardiometabolic outcomes was rated as very low, while evidence for SBP and DBP was rated as low, primarily due to concerns regarding risk of bias and imprecision. Therefore, these findings should be interpreted cautiously, particularly given the limited number of available studies for individual outcomes.

### BMI

Regarding BMI outcomes, the reduction in BMI remained consistent across studies, and the PI remained entirely below the null effect. Nevertheless, the certainty of evidence was rated as low due to concerns regarding risk of bias, suggesting that the magnitude of the observed effect should be interpreted cautiously. The absence of significant differences among the normal weight, overweight, and obesity subgroups suggests that the favorable direction of the BMI effect may not be limited to a particular nutritional status.

The findings of the present review were generally consistent with previous systematic reviews and meta-analyses. Unlike previous reviews that mainly focused on children and adolescents with overweight or obesity, this review included participants with different baseline weight statuses and cardiometabolic health conditions, providing evidence from a broader pediatric population. Hernandez-Martinez et al [29] reported a small but statistically significant reduction in BMI following exergaming interventions among children and adolescents with overweight or obesity. Oliveira et al [25] similarly found small reductions in BMI or BMI z-score at short and intermediate term follow-up, while Comeras-Chueca et al [27] reported favorable effects on both BMI and BMI z-score. Bourke et al [26] reported a significant reduction in BMI z-score among children and adolescents with overweight or obesity, although the pooled effect on absolute BMI did not reach statistical significance. Collectively, these findings suggest that AVG may produce modest improvements in weight-related outcomes, although the magnitude and statistical significance of the effects vary according to the outcome measure and study population.

A network meta‐analysis by Ho et al [23] reported that AVG were more effective than no-AVG comparators in reducing BMI among children and adolescents. A recent umbrella review by Singh et al [58] also found modest improvements in BMI following digital health interventions, which included AVG and serious games. Singh et al [58] also examined whether BMI effects differed according to intervention type. In this review, however, only one study contributed to the VR category [57]. The VR estimate should therefore be regarded as a single-study observation, and the available evidence does not support conclusions regarding the comparative effectiveness of VR and conventional AVG. Singh et al [58] also found that interventions lasting at least 12 weeks produced greater improvements in BMI than shorter interventions, suggesting that intervention duration may be more important than the specific intervention type. Earlier evidence from Biddiss et al [59] showed that AVG could elicit light to moderate intensity physical activity but was insufficient to establish their long-term effectiveness. Together, these findings suggest that intervention dose, sustained engagement, and long-term adherence may be important determinants of BMI-related outcomes.

Several mechanisms may explain the observed effect on BMI. The interactive and enjoyable characteristics of AVG may encourage children and adolescents to participate in physical activity [21]. By increasing energy expenditure during gameplay, repeated AVG participation could contribute to greater overall energy expenditure and a more favorable energy balance [60]. From a practical perspective, AVG may serve as an accessible and engaging adjunct to broader physical activity and weight-management strategies for children and adolescents, rather than as a stand-alone treatment for obesity. Future studies should determine how intervention duration, exercise intensity, game design, behavioral engagement, and adherence support can be optimized to produce larger and more sustainable effects. Longer follow-up periods are also required to establish whether the small changes in BMI observed after AVG translate into meaningful long-term benefits for obesity prevention and weight management.

### Lipid Metabolism and Glucose

#### LDL

The present meta-analysis did not provide clear evidence that AVG reduced LDL in children and adolescents. This finding is consistent with the meta-analysis by Hernandez-Martinez et al [29], which likewise reported no statistically significant improvement in LDL among children and adolescents with overweight or obesity. According to the National Heart, Lung, and Blood Institute (NHLBI) guidelines [61], normal LDL levels for children and adolescents should be below 110 mg/dL. Among the studies included in this analysis, baseline mean LDL levels ranged from 86.4 to 131.6 mg/dL, generally falling within the normal range. But the study by Abedelmalek et al [44] reported a mean baseline LDL level of 131.2‐131.6 mg/dL, which constitutes a high LDL level. And the baseline LDL level in the intervention group of Murphy et al [54] was 111.2 mg/dL, indicating borderline elevation. Some studies may have limited room for improvement, which could attenuate the overall intervention effect of AVG on LDL levels. Dietary intake may also have influenced the observed effects because LDL is sensitive to dietary composition and overall dietary patterns [62]. Most included trials did not consistently control or report participants’ dietary intake during the intervention, making it difficult to distinguish the effects of AVG from those of concurrent dietary changes.

#### HDL

The present analysis found no evidence that AVG significantly improved HDL levels in children and adolescents. This finding is consistent with the meta-analysis by Hernandez-Martinez et al [29], which likewise reported no statistically significant overall improvement in HDL following exergaming interventions among children and adolescents with overweight or obesity. Sensitivity analyses did not alter this conclusion. Several factors may explain the absence of a clear HDL response, including exercise dose, dietary intake, and pubertal development [63-66]. The exercise dose provided in some included trials may have been insufficient to induce measurable changes in HDL, as recent pediatric evidence [66] suggests that lipid responses may depend on session duration, weekly frequency, and total intervention duration. In addition, the age range across the included studies covered different stages of growth and puberty, during which HDL levels may vary physiologically [63,64]. Dietary intake may also have contributed to variation in HDL, as dietary interventions have been shown to modify HDL levels in children and adolescents [65]. However, reports in the included studies regarding pubertal status and dietary intake are relatively scarce, which may obscure the subtle effects of interventions on HDL.

#### Triglycerides

This study found no evidence of improvement in triglycerides levels among children and adolescents following AVG. Similarly, Hernandez-Martinez et al [29] did not observe a significant improvement in triglycerides levels following an AVG in children and adolescents. Exercise training has been shown to increase skeletal muscle lipoprotein lipase (LPL) activity, and exercise-induced changes in muscle LPL activity were inversely associated with changes in plasma triglycerides concentrations [67]. More recent evidence further supports this mechanism, suggesting that skeletal muscle LPL helps break down circulating triglycerides during exercise and facilitates the uptake of triglycerides-derived fatty acids by muscle [68]. Adequately dosed traditional aerobic exercise has been shown to improve triglycerides levels [69]. Whether AVG provide a sufficient exercise stimulus to induce a comparable LPL-mediated adaptation in children and adolescents remains unclear. AVG may provide a relatively modest exercise stimulus [70], which may limit metabolic adaptations related to lipid clearance. The intensity and volume of exercise achieved through AVG may be insufficient to elicit similar metabolic adaptations, which could explain the lack of significant effects observed in our analysis. Additionally, triglycerides responses are sensitive to acute dietary intake, with the type and composition of foods consumed influencing the magnitude of the response [71]. In children and adolescents, Park et al [72] similarly showed that triglycerides concentrations increased significantly after a high-fat meal, whereas a single AVG session did not significantly attenuate the triglycerides response. Therefore, improvements in triglycerides levels may depend on multiple factors, including intervention dosage, baseline triglycerides levels, and dietary habits. This analysis included a limited number of studies, and the study estimates showed some inconsistency (*I*^2^=58%), these findings should be interpreted with caution.

#### TC

This study found that the TC did not show a significant change. The wide prediction interval and the observed inconsistency among study estimates (*I*^2^=87%) suggest considerable uncertainty regarding the effect of AVG across different settings. In the sensitivity analyses, *I*^2^ decreased from 87% to 0% after excluding the study by Abedelmalek et al [44]. This heterogeneity may partly be attributable to differences in participant characteristics, as Abedelmalek et al [44] included participants with severe obesity. Additionally, the intervention in Abedelmalek et al [44] was conducted during Ramadan, and dietary patterns during this period may have influenced participants’ blood lipid levels, causing the effect size for TC to deviate from that of other studies. The intervention period in the study by Abedelmalek et al [44] was also the shortest among all studies, lasting only 4 weeks. The relatively short intervention duration may have limited the potential to detect changes in TC levels. A review by Erçelik et al [28] indicated that AVG can significantly reduce TC levels in overweight or obese children. However, the results of this meta-analysis showed no significant change in TC, which contradicts the conclusions of that review. Differences in study selection criteria, included populations, and the limited number of available trials may partly explain the discrepancy between findings. Because TC reflects the combined contribution of LDL, HDL, and very-low-density lipoprotein cholesterol (VLDL) [73], the absence of significant changes in LDL and HDL may have contributed to the lack of improvement in overall TC levels. Furthermore, the number of studies included in this analysis was too small to support definitive conclusions, limiting the study to an exploratory nature.

#### Glucose

The number of studies and participants included in the analysis of glucose was insufficient, resulting in limited statistical power. No significant improvement was observed, and the interpretation of the current evidence remains subject to considerable uncertainty. Furthermore, the glucose-regulating effect of exercise is primarily achieved through increased glucose uptake by skeletal muscle and improved insulin sensitivity [74]. During exercise, skeletal muscle contraction activates the glucose transporter type 4 (GLUT4) [75], facilitating its translocation from intracellular storage sites to the cell membrane, thereby promoting glucose entry into muscle cells [76,77]. Yin et al [78] suggested that repeated muscle activation throughout the day may contribute to improved glycemic regulation. However, the magnitude of exercise-induced glucose uptake is highly dependent on exercise intensity, frequency, and duration [79]. Although AVG can increase energy expenditure and physical activity levels [80,81], the exercise stimulus may not consistently reach the intensity or duration required to induce substantial metabolic adaptations. Current evidence remains insufficient to confirm a significant beneficial effect of AVG on glucose levels. Further exploration is needed through high-quality studies with larger sample sizes.

#### Blood Pressure and Heart Rate

AVG resulted in a small reduction in SBP, suggesting a potential beneficial effect on blood pressure regulation. However, the magnitude of the effect was limited, and the CI approached the null value, indicating uncertainty regarding the clinical relevance of this finding. This influence may partly reflect the methodological limitations of Christison et al [46], including substantial missing outcome data and reduced completeness of cardiovascular measurements, which may have increased uncertainty in the estimated intervention effect. Zhu et al [30] reported significant reductions in SBP following AVG among children and adolescents under 18 years of age; our findings showed only a marginal reduction. This discrepancy may be explained by differences in the number of included studies, participant characteristics, intervention protocols, and comparator conditions. In contrast, AVG did not significantly affect DBP. The wide CI indicates considerable uncertainty around the estimated effect, which may be attributed to the small number of included studies and limited statistical power rather than the absence of a true effect.

Exercise intensity may be an important determinant of blood pressure responses, although the overall effect may also depend on session duration and accumulated exercise dose [82,83]. The review by Hernandez-Martinez et al [29] also indicates that intensity is a key factor in improving blood pressure. Yin et al [84] found that low-volume high-intensity interval training (LV-HIIT) significantly reduced SBP and DBP compared with nontraining control conditions, despite requiring substantially less exercise time than traditional training modalities. These findings suggest that brief high-intensity exercise may elicit blood pressure adaptations even when total exercise volume is limited. However, LV-HIIT did not produce significantly greater reductions in SBP or DBP than moderate-intensity continuous training or higher-volume HIIT, and these findings therefore do not establish a specific intensity threshold for blood pressure improvement. A review by Tozo et al [85] also indicates that high-intensity interval training (HIIT) interventions with the highest intensity yield the greatest reductions in blood pressure, specifically 3 sessions per week, each lasting 60 minutes, for at least 12 weeks. Previous evidence from broader digital interventions [86], including AVG and exergaming, has shown limited efficacy in improving cardiometabolic health, particularly regarding blood pressure and blood lipids. AVG may provide a modest stimulus for SBP reduction, but the available evidence remains insufficient to determine the intervention intensity, session duration, or accumulated dose required to produce consistent blood pressure adaptations. Future trials should therefore report and systematically evaluate intervention intensity, minutes per session, weekly frequency, adherence, and baseline blood pressure in children and adolescents.

Neither of the 2 studies included in this HR analysis showed a significant between-group effect of HR. In Christison et al [46], HR decreased in both the exergaming and CGs, resulting in no significant between-group difference. Similarly, Wang et al [57] observed reductions in HR in both the REVERIE and CGs after the 8-week intervention, without a significant difference between groups. Thus, the absence of a significant pooled effect may reflect a small AVG-specific effect combined with the very limited evidence base rather than clear evidence that AVG do not influence HR.

The effect of AVG on HR remains uncertain. Only 2 studies contributed to this outcome, and the extremely wide CI and PI indicate considerable uncertainty regarding the magnitude and direction of the effect. The certainty of evidence was rated as very low due to concerns regarding risk of bias and imprecision. Further high-quality trials with larger samples and longer intervention periods are needed to clarify whether AVG can induce meaningful adaptations in cardiac autonomic regulation.

### Limitations and Future Research

The limitations of this systematic review and meta-analysis include: (1) Several outcomes were supported by small numbers of studies and participants, resulting in imprecise effect estimates and limited statistical power. In addition, the small numbers of studies and participants within some BMI subgroups restricted the robustness of the findings regarding potential moderator effects, including sex, intervention type, and nutritional status; (2) The included studies differed in terms of intervention equipment and game scenarios, which may also have influenced the effectiveness of the interventions; (3) Due to the limited number of studies included in this review and the lack of clear reporting on exercise protocols and exercise intensity, meta-regression analysis was not conducted; (4) Inconsistencies in intervention dose across the included studies may have led to between-study variability in results; (5) This review included only published studies with accessible full−text articles, and it did not search for gray literature or unpublished data, which may have led to publication bias in the results. This concern is particularly relevant because few studies contributed to most outcomes, limiting the reliability of formal assessments of small-study effects; (6) Although BMI was the most consistently reported anthropometric outcome across the included studies, raw BMI does not fully account for age, sex, and maturation-related changes in children and adolescents, which may limit the accuracy of weight-status interpretation.

Therefore, future research should include more high-quality, large-scale studies to validate these findings. These studies should explicitly report exercise intensity parameters and appropriately extend the intervention duration to determine whether AVG can effectively improve cardiometabolic health in children and adolescents when administered at a higher intervention dose. The interactivity and fun elements of AVG may help promote initial engagement and intervention adherence. However, existing studies report inconsistent findings regarding the assessment of engagement-related indicators, and the impact of AVG on long-term engagement behavior remains unclear. Future research should use standardized, objectively measurable tools for assessing engagement, and prospectively evaluate whether perceived fun translates into sustained engagement behavior.

### Conclusion

This systematic review and meta-analysis provide an updated and broader RCT-only evaluation of the effects of AVG on cardiometabolic health in children and adolescents, including anthropometric, cardiovascular, and metabolic outcomes. In contrast to previous reviews that have focused on selected populations or narrower outcome domains, this review evaluates a broader pediatric population and a wider range of cardiometabolic outcomes within a unified framework. The findings suggest that AVG may be associated with small reductions in BMI and SBP, although the SBP effect was less consistent across potential future settings, and evidence for other cardiometabolic outcomes remains uncertain. By clarifying both the potential benefits and the remaining evidence gaps, this review provides a more comprehensive basis for future research and intervention development. From a practical perspective, AVG may serve as an engaging adjunct to support physical activity participation among children and adolescents. Further high-quality RCTs are needed to determine the optimal intervention characteristics and clarify their long-term cardiometabolic effects.

## Supplementary material

10.2196/101833 Multimedia Appendix 1 Supplementary search results, funnel plot, leave-one-out sensitivity analysis, GRADE (Grading of Recommendations Assessment, Development and Evaluation) summary of findings, and Physiotherapy Evidence Database (PEDro) methodological quality assessment.

10.2196/101833 Checklist 1 PRISMA 2020 checklist.

10.2196/101833 Checklist 2 PRISMA 2020 for Abstracts checklist.

## Abbreviations

AVG: active video games
BMI-z: body mass index z score
CG: control group
DBP: diastolic blood pressure
DTC: delayed-treatment control group
EDF: endothelial dysfunction
EX: experimental group
GLUT 4: glucose transporter type 4
GRADE: Grading of Recommendations Assessment, Development and Evaluation
HBA_1c_: glycated hemoglobin
HDL: high-density lipoprotein cholesterol
HIIT: high-intensity interval training
HR: heart rate
LDL: low-density lipoprotein cholesterol
LPL: lipoprotein lipase
LV−HIIT: low-volume high-intensity interval training
NHLBI: National Heart, Lung, and Blood Institute
PEDro: Physiotherapy Evidence Database
PI: prediction interval
PICOS: Population, intervention, comparison, outcomes, and study design
PRISMA: Preferred Reporting Items for Systematic Reviews and Meta-Analyses
PRISMA-S: Preferred Reporting Items for Systematic Reviews and Meta-Analyses literature search extension
PROSPERO: International Prospective Register of Systematic Reviews
RCTs: randomized controlled trials
RoB 2: Cochrane Risk of Bias 2 tool
SBP: systolic blood pressure
TC: total cholesterol
VLDL: very-low-density lipoprotein cholesterol
VR: virtual reality

## References

[1] Barnett TA, Kelly AS, Young DR (2018). Sedentary behaviors in today’s youth: approaches to the prevention and management of childhood obesity: a scientific statement from the American Heart Association. Circulation.

[2] Georgoulis M, Grapsa I, Arnaoutis G (2026). A systematic review and meta-analysis of longitudinal studies exploring the link between physical inactivity and indicators of childhood overweight/obesity and metabolically unhealthy obesity risk in Western countries. Curr Obes Rep.

[3] Tremblay MS, LeBlanc AG, Kho ME (2011). Systematic review of sedentary behaviour and health indicators in school-aged children and youth. Int J Behav Nutr Phys Act.

[4] Agbaje AO, Perng W, Tuomainen TP (2023). Effects of accelerometer-based sedentary time and physical activity on DEXA-measured fat mass in 6059 children. Nat Commun.

[5] Harnois-Leblanc S, Sylvestre MP, Van Hulst A (2023). Estimating causal effects of physical activity and sedentary behaviours on the development of type 2 diabetes in at-risk children from childhood to late adolescence: an analysis of the QUALITY cohort. Lancet Child Adolesc Health.

[6] Jebeile H, Kelly AS, O’Malley G, Baur LA (2022). Obesity in children and adolescents: epidemiology, causes, assessment, and management. Lancet Diabetes Endocrinol.

[7] Smith JJ, Eather N, Morgan PJ, Plotnikoff RC, Faigenbaum AD, Lubans DR (2014). The health benefits of muscular fitness for children and adolescents: a systematic review and meta-analysis. Sports Med.

[8] Skinner AC, Perrin EM, Moss LA, Skelton JA (2015). Cardiometabolic risks and severity of obesity in children and young adults. N Engl J Med.

[9] Thompson-Paul AM, Kraus EM, Porter RM (2024). Pediatric lipid screening prevalence using nationwide electronic medical records. JAMA Netw Open.

[10] Friedemann C, Heneghan C, Mahtani K, Thompson M, Perera R, Ward AM (2012). Cardiovascular disease risk in healthy children and its association with body mass index: systematic review and meta-analysis. BMJ.

[11] Nadeau KJ, Maahs DM, Daniels SR, Eckel RH (2011). Childhood obesity and cardiovascular disease: links and prevention strategies. Nat Rev Cardiol.

[12] Chen H, Chen C, Spanos M (2022). Exercise training maintains cardiovascular health: signaling pathways involved and potential therapeutics. Signal Transduct Target Ther.

[13] Momma H, Kawakami R, Honda T, Sawada SS (2022). Muscle-strengthening activities are associated with lower risk and mortality in major non-communicable diseases: a systematic review and meta-analysis of cohort studies. Br J Sports Med.

[14] Moreira JBN, Wohlwend M, Wisløff U (2020). Exercise and cardiac health: physiological and molecular insights. Nat Metab.

[15] Valenzuela PL, Ruilope LM, Santos-Lozano A (2023). Exercise benefits in cardiovascular diseases: from mechanisms to clinical implementation. Eur Heart J.

[16] van Sluijs EMF, Ekelund U, Crochemore-Silva I (2021). Physical activity behaviours in adolescence: current evidence and opportunities for intervention. Lancet.

[17] Han A, Fu A, Cobley S, Sanders RH (2018). Effectiveness of exercise intervention on improving fundamental movement skills and motor coordination in overweight/obese children and adolescents: a systematic review. J Sci Med Sport.

[18] Pearl RL, Wadden TA, Jakicic JM (2021). Is weight stigma associated with physical activity? A systematic review. Obesity (Silver Spring).

[19] Stankov I, Olds T, Cargo M (2012). Overweight and obese adolescents: what turns them off physical activity?. Int J Behav Nutr Phys Act.

[20] Gao Z, Chen S, Pasco D, Pope Z (2015). A meta-analysis of active video games on health outcomes among children and adolescents. Obes Rev.

[21] Moller AC, Sousa CV, Lee KJ, Alon D, Lu AS (2023). Active video game interventions targeting physical activity behaviors: systematic review and meta-analysis. J Med Internet Res.

[22] Best JR (2013). Exergaming in youth: effects on physical and cognitive health. Z Psychol.

[23] Ho RST, Chan EKY, Liu KKY, Wong SHS (2022). Active video game on children and adolescents’ physical activity and weight management: a network meta-analysis. Scand J Med Sci Sports.

[24] Wang M, Xu J, Zhou X, Li X, Zheng Y (2025). Effectiveness of gamification interventions to improve physical activity and sedentary behavior in children and adolescents: systematic review and meta-analysis. JMIR Serious Games.

[25] Oliveira CB, Pinto RZ, Saraiva BTC (2020). Effects of active video games on children and adolescents: a systematic review with meta-analysis. Scand J Med Sci Sports.

[26] Bourke M, Patterson L, Di Nardo F, Whittaker P, Verma A (2023). Active video games and weight management in overweight children and adolescents-systematic review and meta-analysis. J Public Health (Oxf).

[27] Comeras-Chueca C, Marin-Puyalto J, Matute-Llorente A, Vicente-Rodriguez G, Casajus JA, Gonzalez-Aguero A (2021). Effects of active video games on health-related physical fitness and motor competence in children and adolescents with overweight or obesity: systematic review and meta-analysis. JMIR Serious Games.

[28] Erçelik ZE, Çağlar S (2022). Effectiveness of active video games in overweight and obese adolescents: a systematic review and meta-analysis of randomized controlled trials. Ann Pediatr Endocrinol Metab.

[29] Hernandez-Martinez J, Perez-Carcamo J, Melki H (2024). Effects of exergaming on morphological variables, biochemical parameters, and blood pressure in children and adolescents with overweight/obesity: a systematic review with meta-analysis of randomized controlled trials. Children (Basel).

[30] Zhu H, Tung KTS, So HK (2025). Role of active video games in blood pressure management among children and young adults: systematic review and meta-analysis. J Med Internet Res.

[31] Page MJ, McKenzie JE, Bossuyt PM (2021). The PRISMA 2020 statement: an updated guideline for reporting systematic reviews. BMJ.

[32] Rethlefsen ML, Kirtley S, Waffenschmidt S (2021). PRISMA-S: an extension to the PRISMA statement for reporting literature searches in systematic reviews. Syst Rev.

[33] Drevon D, Fursa SR, Malcolm AL (2017). Intercoder reliability and validity of WebPlotDigitizer in extracting graphed data. Behav Modif.

[34] Higgins JPT, Thomas J, Chandler J (2019). Cochrane Handbook for Systematic Reviews of Interventions.

[35] Sterne JAC, Savović J, Page MJ (2019). RoB 2: a revised tool for assessing risk of bias in randomised trials. BMJ.

[36] de Morton NA (2009). The PEDro scale is a valid measure of the methodological quality of clinical trials: a demographic study. Aust J Physiother.

[37] Viechtbauer W (2010). Conducting meta-analyses in r with the metafor package. J Stat Softw.

[38] IntHout J, Ioannidis JPA, Borm GF (2014). The Hartung-Knapp-Sidik-Jonkman method for random effects meta-analysis is straightforward and considerably outperforms the standard DerSimonian-Laird method. BMC Med Res Methodol.

[39] Brini S, Leung TI (2026). Value and credibility of meta-analysis: tutorial on enhancing methodological rigor and AI-powered efficiency. J Med Internet Res.

[40] Nagashima K, Noma H, Furukawa TA (2019). Prediction intervals for random-effects meta-analysis: a confidence distribution approach. Stat Methods Med Res.

[41] Higgins JPT, López-López JA (2026). Reflections on the I-squared index for measuring inconsistency in meta-analysis. Res Synth Methods.

[42] Sterne JAC, Sutton AJ, Ioannidis JPA (2011). Recommendations for examining and interpreting funnel plot asymmetry in meta-analyses of randomised controlled trials. BMJ.

[43] Guyatt GH, Oxman AD, Vist GE (2008). GRADE: an emerging consensus on rating quality of evidence and strength of recommendations. BMJ.

[44] Abedelmalek S, Aloui K, Denguezli Bouzgarou M, Adam H, Souissi N, Chtourou H (2022). Exergaming during Ramadan intermittent fasting improve body composition as well as physiological and psychological responses to physical exercise in adolescents with obesity. Front Nutr.

[45] Berg J, Wang AI, Madssen E, Røe L, Moholdt T (2026). Effects of providing access to a cycling exergaming platform on cardiorespiratory fitness and cardiometabolic health in adolescents: a randomised, controlled trial. Sci Rep.

[46] Christison AL, Evans TA, Bleess BB, Wang H, Aldag JC, Binns HJ (2016). Exergaming for health: a randomized study of community-based exergaming curriculum in pediatric weight management. Games Health J.

[47] Coknaz D, Mirzeoglu AD, Atasoy HI, Alkoy S, Coknaz H, Goral K (2019). A digital movement in the world of inactive children: favourable outcomes of playing active video games in a pilot randomized trial. Eur J Pediatr.

[48] Comeras-Chueca C, Villalba-Heredia L, Perez-Lasierra JL (2022). Effect of an active video game intervention combined with multicomponent exercise for cardiorespiratory fitness in children with overweight and obesity: randomized controlled trial. JMIR Serious Games.

[49] Gao Z, Lee JE, Zeng N, Pope ZC, Zhang Y, Li X (2019). Home-based exergaming on preschoolers’ energy expenditure, cardiovascular fitness, body mass index and cognitive flexibility: a randomized controlled trial. J Clin Med.

[50] Lau PWC, Wang JJ, Maddison R (2016). A randomized-controlled trial of school-based active videogame intervention on chinese children’s aerobic fitness, physical activity level, and psychological correlates. Games Health J.

[51] Lu AS, Baranowski T, Barreira TV (2025). The impact of narratives and active video games among black and hispanic children with overweight and obesity: a randomized controlled trial. Int J Behav Nutr Phys Act.

[52] Maddison R, Foley L, Ni Mhurchu C (2011). Effects of active video games on body composition: a randomized controlled trial. Am J Clin Nutr.

[53] Marsigliante S, My G, Mazzotta G, Muscella A (2024). The effects of exergames on physical fitness, body composition and enjoyment in children: a six-month intervention study. Children (Basel).

[54] Murphy ECS, Carson L, Neal W, Baylis C, Donley D, Yeater R (2009). Effects of an exercise intervention using Dance Dance Revolution on endothelial function and other risk factors in overweight children. Int J Pediatr Obes.

[55] Staiano AE, Beyl RA, Guan W, Hendrick CA, Hsia DS, Newton RL (2018). Home-based exergaming among children with overweight and obesity: a randomized clinical trial. Pediatr Obes.

[56] Staiano AE, Marker AM, Beyl RA, Hsia DS, Katzmarzyk PT, Newton RL (2017). A randomized controlled trial of dance exergaming for exercise training in overweight and obese adolescent girls. Pediatr Obes.

[57] Wang J, Qin Y, Wu Q (2025). An adaptive AI-based virtual reality sports system for adolescents with excess body weight: a randomized controlled trial. Nat Med.

[58] Singh B, Ahmed M, Staiano AE (2025). Lifestyle eHealth and mHealth interventions for children and adolescents: systematic umbrella review and meta-meta-analysis. J Med Internet Res.

[59] Biddiss E, Irwin J (2010). Active video games to promote physical activity in children and youth: a systematic review. Arch Pediatr Adolesc Med.

[60] Spring FDH, Lærkholm G, Jensen RB, Kloppenborg JT (2025). The effect of exergaming on BMI and fitness in children and adolescents with obesity: a systematic review. Acta Paediatr.

[61] Expert Panel on Integrated Guidelines for Cardiovascular Health and Risk Reduction in Children and Adolescents, National Heart, Lung, and Blood Institute (2011). Expert panel on integrated guidelines for cardiovascular health and risk reduction in children and adolescents: summary report. Pediatrics.

[62] Gomez-Delgado F, Katsiki N, Lopez-Miranda J, Perez-Martinez P (2021). Dietary habits, lipoprotein metabolism and cardiovascular disease: from individual foods to dietary patterns. Crit Rev Food Sci Nutr.

[63] Bouhour S, Plantefève R, Gillet V (2024). Establishing non-fasting reference values for plasma lipids levels based on age, sex, and puberty stage in a French-Canadian pediatric population. Lipids Health Dis.

[64] Eissa MA, Mihalopoulos NL, Holubkov R, Dai S, Labarthe DR (2016). Changes in Fasting Lipids during Puberty. J Pediatr.

[65] López-Gil JF, García-Hermoso A, Martínez-González MÁ, Rodríguez-Artalejo F (2024). Mediterranean diet and cardiometabolic biomarkers in children and adolescents: a systematic review and meta-analysis. JAMA Netw Open.

[66] Men J, Zhu G, Li Y (2025). Impact of exercise on cardiovascular disease risk in overweight or obese children and adolescents: a systematic review and meta-analysis. BMC Sports Sci Med Rehabil.

[67] Seip RL, Angelopoulos TJ, Semenkovich CF (1995). Exercise induces human lipoprotein lipase gene expression in skeletal muscle but not adipose tissue. Am J Physiol.

[68] Johnsson K, George JG, Parks EJ, Katsanos CS (2025). Obesity versus endurance exercise training: plasma triacylglycerol and muscle lipoprotein lipase activity at the crossroads of lipid accumulation in muscle. Am J Physiol Endocrinol Metab.

[69] Liu Q, Xin X, Wang H (2026). Effects of different exercise modalities on lipid profiles in overweight and obese children and adolescents: a systematic review and network meta-analysis of randomized controlled trials. BMC Sports Sci Med Rehabil.

[70] Peng W, Lin JH, Crouse J (2011). Is playing exergames really exercising? A meta-analysis of energy expenditure in active video games. Cyberpsychol Behav Soc Netw.

[71] Lee DPS, Low JHM, Chen JR, Zimmermann D, Actis-Goretta L, Kim JE (2020). The influence of different foods and food ingredients on acute postprandial triglyceride response: a systematic literature review and meta-analysis of randomized controlled trials. Adv Nutr.

[72] Park SH, Yoon ES, Lee YH (2015). Effects of acute active video games on endothelial function following a high-fat meal in overweight adolescents. J Phys Act Health.

[73] Kwiterovich PO (2000). The metabolic pathways of high-density lipoprotein, low-density lipoprotein, and triglycerides: a current review. Am J Cardiol.

[74] Richter EA, Bilan PJ, Klip A (2025). A comprehensive view of muscle glucose uptake: regulation by insulin, contractile activity, and exercise. Physiol Rev.

[75] Borghouts LB, Keizer HA (2000). Exercise and insulin sensitivity: a review. Int J Sports Med.

[76] Iaccarino G, Franco D, Sorriento D, Strisciuglio T, Barbato E, Morisco C (2021). Modulation of insulin sensitivity by exercise training: implications for cardiovascular prevention. J Cardiovasc Transl Res.

[77] Lund S, Holman GD, Schmitz O, Pedersen O (1995). Contraction stimulates translocation of glucose transporter GLUT4 in skeletal muscle through a mechanism distinct from that of insulin. Proc Natl Acad Sci U S A.

[78] Yin M, Xu K, Deng J (2024). Optimal frequency of interrupting prolonged sitting for cardiometabolic health: a systematic review and meta-analysis of randomized crossover trials. Scand J Med Sci Sports.

[79] Richter EA, Hargreaves M (2013). Exercise, GLUT4, and skeletal muscle glucose uptake. Physiol Rev.

[80] Gao Z, Pope Z, Lee JE (2017). Impact of exergaming on young children’s school day energy expenditure and moderate-to-vigorous physical activity levels. J Sport Health Sci.

[81] Smith JL, Carbine KA, Larson MJ (2021). To play or not to play? The relationship between active video game play and electrophysiological indices of food-related inhibitory control in adolescents. Eur J Neurosci.

[82] Yuan H, Shao W, Ren Y, Jiang M, Kong J, Jing F (2025). Non-linear effect of physical activity intervention on blood pressure in children and adolescents: a systematic review and dose-response meta-analysis. BMC Public Health.

[83] Zhou J, Sun W, Tang S (2025). Effects of exercise interventions on blood pressure in children and adolescents with overweight or obesity: a systematic review and meta-analysis of randomized controlled trials. J Adolesc Health.

[84] Yin M, Li H, Bai M (2024). Is low-volume high-intensity interval training a time-efficient strategy to improve cardiometabolic health and body composition? A meta-analysis. Appl Physiol Nutr Metab.

[85] Tozo JVA, Tadiotto MC, Tozo TAA (2025). Effects of different physical exercise programs on blood pressure in overweight children and adolescents: systematic review and meta-analysis. BMC Pediatr.

[86] Palacios C, Hernandez J, Ajmal A (2025). Digital health, technology-driven or technology-assisted interventions for the management of obesity in children and adolescents. Cochrane Database Syst Rev.

