# Supplementary material for: Effects of Active Video Games on Cardiometabolic Health in Children and Adolescents: Systematic Review and Meta-Analysis of Randomized Controlled Trials
Source: JMIR Serious Games. 2026 Oct 13;14:e101833. doi: 10.2196/101833 (PMC13660585; doi:10.2196/101833)
Supplement: Supplementary file 1 [file games-v14-e101833-s001.docx]

**Electronic Supplementary Material Contents**

| **Number** | **Material** | **Page** |
| --- | --- | --- |
| 1 | Electronic Supplementary Material Appendix S2 (Search results) | 2-5 |
| 2 | Electronic Supplementary Material Appendix S3 (Funnel plot) | 6 |
| 3 | Electronic Supplementary Material Appendix S4 (Leave-one-out) | 7-9 |
| 4 | Electronic Supplementary Material Appendix S5 (GRADE Summary of Findings Table) | 10-12 |
| 5 | Electronic Supplementary Material Appendix S6 (Methodological quality assessment [PEDro]) | 13-14 |

**Electronic Supplementary Material Appendix S2 (Search Results)**

| **Databases** | **Query** | **Results** |
| --- | --- | --- |
| **Pubmed** | ("Exergaming"[Mesh] OR "Video Games"[Mesh] OR exergam*[Title/Abstract] OR exer-gam*[Title/Abstract] OR "active video game"[Title/Abstract] OR "active video games"[Title/Abstract] OR "active videogame"[Title/Abstract] OR "active videogames"[Title/Abstract] OR "physically active video game"[Title/Abstract] OR "physically active video games"[Title/Abstract] OR "exercise video game"[Title/Abstract] OR "exercise video games"[Title/Abstract] OR "interactive video game"[Title/Abstract] OR "interactive video games"[Title/Abstract] OR "motion-controlled game"[Title/Abstract] OR "motion-controlled games"[Title/Abstract] OR "movement-based game"[Title/Abstract] OR "movement-based games"[Title/Abstract] OR "active gaming"[Title/Abstract] OR "gamified exercise"[Title/Abstract] OR "virtual reality game"[Title/Abstract] OR "virtual reality games"[Title/Abstract] OR Wii[Title/Abstract] OR "Wii Fit"[Title/Abstract] OR Kinect*[Title/Abstract] OR Xbox*[Title/Abstract] OR "PlayStation Move"[Title/Abstract] OR "Nintendo Switch Sports"[Title/Abstract] OR "Dance Dance Revolution"[Title/Abstract] OR EyeToy[Title/Abstract]) AND ("Child, Preschool"[Mesh] OR "Child"[Mesh] OR "Adolescent"[Mesh] OR child*[Title/Abstract] OR adolescen*[Title/Abstract] OR teen*[Title/Abstract] OR youth*[Title/Abstract] OR pediatric*[Title/Abstract] OR paediatric*[Title/Abstract] OR schoolchild*[Title/Abstract] OR "school child"[Title/Abstract] OR "school children"[Title/Abstract] OR "school-aged"[Title/Abstract] OR "school age"[Title/Abstract] OR "high school student"[Title/Abstract] OR "high school students"[Title/Abstract] OR "young person"[Title/Abstract] OR "young people"[Title/Abstract]) AND ("Cardiometabolic Risk Factors"[Mesh] OR "Metabolic Syndrome"[Mesh] OR "Blood Pressure"[Mesh] OR "Hypertension"[Mesh] OR "Blood Glucose"[Mesh] OR "Insulin Resistance"[Mesh] OR "Cholesterol"[Mesh] OR "Triglycerides"[Mesh] OR "Lipoproteins, LDL"[Mesh] OR "Lipoproteins, HDL"[Mesh] OR "Oxygen Consumption"[Mesh] OR "Cardiorespiratory Fitness"[Mesh] OR "Ventricular Function, Left"[Mesh] OR "Stroke Volume"[Mesh] OR "Heart Rate"[Mesh] OR "Body Mass Index"[Mesh] OR "Waist Circumference"[Mesh] OR "Body Composition"[Mesh] OR "Adiposity"[Mesh] OR "Insulin"[Mesh] OR cardiometabolic*[Title/Abstract] OR "cardiometabolic health"[Title/Abstract] OR "metabolic syndrome"[Title/Abstract] OR "metabolic risk"[Title/Abstract] OR "blood pressure"[Title/Abstract] OR hypertension[Title/Abstract] OR "systolic blood pressure"[Title/Abstract] OR "diastolic blood pressure"[Title/Abstract] OR SBP[Title/Abstract] OR DBP[Title/Abstract] OR "blood glucose"[Title/Abstract] OR "fasting glucose"[Title/Abstract] OR glycem*[Title/Abstract] OR glycaem*[Title/Abstract] OR "fasting insulin"[Title/Abstract] OR "insulin resistance"[Title/Abstract] OR "HOMA-IR"[Title/Abstract] OR HOMAIR[Title/Abstract] OR "lipid profile"[Title/Abstract] OR lipid*[Title/Abstract] OR cholesterol*[Title/Abstract] OR triglycerid*[Title/Abstract] OR LDL[Title/Abstract] OR HDL[Title/Abstract] OR "low-density lipoprotein"[Title/Abstract] OR "low density lipoprotein"[Title/Abstract] OR "high-density lipoprotein"[Title/Abstract] OR "high density lipoprotein"[Title/Abstract] OR VO2max[Title/Abstract] OR "VO2 max"[Title/Abstract] OR "maximal oxygen uptake"[Title/Abstract] OR "peak oxygen uptake"[Title/Abstract] OR "peak aerobic capacity"[Title/Abstract] OR "aerobic capacity"[Title/Abstract] OR "cardiorespiratory fitness"[Title/Abstract] OR "cardiorespiratory fitness"[Title/Abstract] OR "left ventricular ejection fraction"[Title/Abstract] OR LVEF[Title/Abstract] OR "stroke volume"[Title/Abstract] OR "heart rate"[Title/Abstract] OR "resting heart rate"[Title/Abstract] OR BMI[Title/Abstract] OR "body mass index"[Title/Abstract] OR "waist circumference"[Title/Abstract] OR "body fat"[Title/Abstract] OR "percent body fat"[Title/Abstract] OR "percentage body fat"[Title/Abstract] OR adiposity[Title/Abstract]) | 597 |
| **Web of Science** | TS=(exergam* OR exer-gam* OR "active video game" OR "active video games" OR "active videogame" OR "active videogames" OR "physically active video game" OR "physically active video games" OR "exercise video game" OR "exercise video games" OR "interactive video game" OR "interactive video games" OR "motion-controlled game" OR "motion-controlled games" OR "movement-based game" OR "movement-based games" OR "active gaming" OR "gamified exercise" OR "virtual reality game" OR "virtual reality games" OR Wii OR "Wii Fit" OR Kinect* OR Xbox* OR "PlayStation Move" OR "Nintendo Switch Sports" OR "Dance Dance Revolution" OR EyeToy) AND TS=(child* OR adolescen* OR teen* OR youth* OR pediatric* OR paediatric* OR schoolchild* OR "school child" OR "school children" OR "school-aged" OR "school age" OR "high school student" OR "high school students" OR "young person" OR "young people") AND TS=(cardiometabolic* OR "cardiometabolic health" OR "metabolic syndrome" OR "metabolic risk" OR "blood pressure" OR hypertension OR "systolic blood pressure" OR "diastolic blood pressure" OR SBP OR DBP OR "blood glucose" OR "fasting glucose" OR glycem* OR glycaem* OR "fasting insulin" OR "insulin resistance" OR "HOMA-IR" OR HOMAIR OR "lipid profile" OR lipid* OR cholesterol* OR triglycerid* OR LDL OR HDL OR "low-density lipoprotein" OR "low density lipoprotein" OR "high-density lipoprotein" OR "high density lipoprotein" OR VO2max OR "VO2 max" OR "maximal oxygen uptake" OR "peak oxygen uptake" OR "peak aerobic capacity" OR "aerobic capacity" OR "cardiorespiratory fitness" OR "cardiorespiratory fitness" OR "left ventricular ejection fraction" OR LVEF OR "stroke volume" OR "heart rate" OR "resting heart rate" OR BMI OR "body mass index" OR "waist circumference" OR "body fat" OR "percent body fat" OR "percentage body fat" OR adiposity) | 294 |
| **Cochrane Library** | ([mh "Exergaming"] OR [mh "Video Games"] OR (exergam* OR exer-gam* OR "active video game" OR "active video games" OR "active videogame" OR "active videogames" OR "physically active video game" OR "physically active video games" OR "exercise video game" OR "exercise video games" OR "interactive video game" OR "interactive video games" OR "motion-controlled game" OR "motion-controlled games" OR "movement-based game" OR "movement-based games" OR "active gaming" OR "gamified exercise" OR "virtual reality game" OR "virtual reality games" OR Wii OR "Wii Fit" OR Kinect* OR Xbox* OR "PlayStation Move" OR "Nintendo Switch Sports" OR "Dance Dance Revolution" OR EyeToy):ti,ab,kw) AND ([mh "Child, Preschool"] OR [mh "Child"] OR [mh "Adolescent"] OR (child* OR adolescen* OR teen* OR youth* OR pediatric* OR paediatric* OR schoolchild* OR "school child" OR "school children" OR "school-aged" OR "school age" OR "high school student" OR "high school students" OR "young person" OR "young people"):ti,ab,kw) AND ([mh "Cardiometabolic Risk Factors"] OR [mh "Metabolic Syndrome"] OR [mh "Blood Pressure"] OR [mh "Hypertension"] OR [mh "Blood Glucose"] OR [mh "Insulin Resistance"] OR [mh "Cholesterol"] OR [mh "Triglycerides"] OR [mh "Lipoproteins, LDL"] OR [mh "Lipoproteins, HDL"] OR [mh "Oxygen Consumption"] OR [mh "Cardiorespiratory Fitness"] OR [mh "Ventricular Function, Left"] OR [mh "Stroke Volume"] OR [mh "Heart Rate"] OR [mh "Body Mass Index"] OR [mh "Waist Circumference"] OR [mh "Body Composition"] OR [mh "Adiposity"] OR [mh "Insulin"] OR (cardiometabolic* OR "cardiometabolic health" OR "metabolic syndrome" OR "metabolic risk" OR "blood pressure" OR hypertension OR "systolic blood pressure" OR "diastolic blood pressure" OR SBP OR DBP OR "blood glucose" OR "fasting glucose" OR glycem* OR glycaem* OR "fasting insulin" OR "insulin resistance" OR "HOMA-IR" OR HOMAIR OR "lipid profile" OR lipid* OR cholesterol* OR triglycerid* OR LDL OR HDL OR "low-density lipoprotein" OR "low density lipoprotein" OR "high-density lipoprotein" OR "high density lipoprotein" OR VO2max OR "VO2 max" OR "maximal oxygen uptake" OR "peak oxygen uptake" OR "peak aerobic capacity" OR "aerobic capacity" OR "cardiorespiratory fitness" OR "cardiorespiratory fitness" OR "left ventricular ejection fraction" OR LVEF OR "stroke volume" OR "heart rate" OR "resting heart rate" OR BMI OR "body mass index" OR "waist circumference" OR "body fat" OR "percent body fat" OR "percentage body fat" OR adiposity):ti,ab,kw) | 178 |
| **Embase** | ('exergaming'/exp OR 'video game'/exp OR "Exergaming":ti,ab,kw OR "Video Games":ti,ab,kw OR "active video game*":ti,ab,kw OR "exergame*":ti,ab,kw OR "exer-game*":ti,ab,kw OR "active gaming":ti,ab,kw OR "Wii":ti,ab,kw OR "Kinect":ti,ab,kw OR "Xbox":ti,ab,kw OR "PlayStation Move":ti,ab,kw OR "Nintendo Switch Sports":ti,ab,kw OR "gamified exercise":ti,ab,kw OR "virtual reality game*":ti,ab,kw) AND ('child'/exp OR 'adolescent'/exp OR "child":ti,ab,kw OR "school children":ti,ab,kw OR "adolescents":ti,ab,kw OR "teen":ti,ab,kw OR "high school student":ti,ab,kw OR "young":ti,ab,kw) AND ('metabolic syndrome x'/exp OR 'blood pressure'/exp OR 'hypertension'/exp OR 'blood glucose'/exp OR 'insulin resistance'/exp OR 'cholesterol'/exp OR 'triglyceride'/exp OR 'low density lipoprotein'/exp OR 'high density lipoprotein'/exp OR 'oxygen consumption'/exp OR 'left ventricular function'/exp OR 'systolic blood pressure'/exp OR 'diastolic blood pressure'/exp OR 'heart rate'/exp OR 'stroke volume'/exp OR 'body mass index'/exp OR 'waist circumference'/exp OR 'body fat'/exp OR 'insulin'/exp OR "Cardiometabolic Risk Factors":ti,ab,kw OR "Metabolic Syndrome":ti,ab,kw OR "Cardiometabolic health":ti,ab,kw OR "metabolic risk":ti,ab,kw OR "blood pressure":ti,ab,kw OR "hypertension":ti,ab,kw OR "blood glucose":ti,ab,kw OR "insulin resistance":ti,ab,kw OR "HOMA-IR":ti,ab,kw OR "lipid profile":ti,ab,kw OR "cholesterol":ti,ab,kw OR "triglycerides":ti,ab,kw OR "LDL":ti,ab,kw OR "HDL":ti,ab,kw OR "VO2max ":ti,ab,kw OR "peak aerobic capacity":ti,ab,kw OR "VO2":ti,ab,kw OR "left ventricular ejection fraction":ti,ab,kw OR "systolic":ti,ab,kw OR "diastolic":ti,ab,kw OR "resting heart rate":ti,ab,kw OR "stroke volume":ti,ab,kw OR "body mass index":ti,ab,kw OR "waist circumference":ti,ab,kw OR "percentage body fat":ti,ab,kw OR "fasting insulin":ti,ab,kw) | 1232 |

**Electronic Supplementary Material Appendix S3 (Funnel plot)**

**BMI**

**Electronic Supplementary Material Appendix S4 (A Sensitivity Analysis based on Leave-one-out)**

**BMI**

**SBP**

**DBP**

**TC**

**TG**

**HDL**

**LDL**

**HR**

**Glucose**

**Electronic Supplementary Material Appendix S5 (GRADE Summary of Findings Table)**

| **Summary of findings:** | | | | | |
| --- | --- | --- | --- | --- | --- |
| **Active video games compared to Control (no intervention, sedentary video games, or regular physical activity) for Children and adolescents aged 4–18 years** | | | | | |
| **Patient or population:** Children and adolescents aged 4–18 years  **Setting:**  **Intervention:** Active video games  **Comparison:** Control (no intervention, sedentary video games, or regular physical activity) | | | | | |
| Outcomes | **Anticipated absolute effects^*^** (95% CI) | | Relative effect (95% CI) | № of participants (studies) | Certainty of the evidence (GRADE) |
|  | **Risk with Control (no intervention, sedentary video games, or regular physical activity)** | **Risk with Active video games** |  |  |  |
| BMI | - | SMD **0.23 SD lower** (0.35 lower to 0.10 lower) | - | 899 (11 RCTs) | ⨁⨁◯◯ Low^a^ |
| SBP | - | SMD **0.18 SD lower** (0.36 lower to 0.001 lower) | - | 243 (4 RCTs) | ⨁⨁◯◯ Low^b^ |
| DBP | - | SMD **0.11 SD lower** (0.86 lower to 0.63 higher) | - | 195 (3 RCTs) | ⨁⨁◯◯ Low^c,d^ |
| TC | - | SMD **0.96 SD lower** (3.79 lower to 1.88 higher) | - | 208 (4 RCTs) | ⨁◯◯◯ Very low^e,f,g^ |
| TG | - | SMD **0.20 SD lower** (0.70 lower to 0.31 higher) | - | 342 (7 RCTs) | ⨁◯◯◯ Very low^h,i,j^ |
| LDL | - | SMD **0.26 SD lower** (0.64 lower to 0.13 higher) | - | 342 (7 RCTs) | ⨁◯◯◯ Very low^k,l^ |
| HDL | - | SMD **0.05 SD higher** (0.21 lower to 0.31 higher) | - | 342 (7 RCTs) | ⨁◯◯◯ Very low^m,n^ |
| HR | - | SMD **0.14 SD lower** (1.74 lower to 1.46 higher) | - | 186 (2 RCTs) | ⨁◯◯◯ Very low^h,o^ |
| Glucose | - | SMD **0.17 SD lower** (0.37 lower to 0.02 higher) | - | 180 (5 RCTs) | ⨁◯◯◯ Very low^h,p^ |
| ***The risk in the intervention group** (and its 95% confidence interval) is based on the assumed risk in the comparison group and the **relative effect** of the intervention (and its 95% CI).  **CI:** confidence interval; **SMD:** standardised mean difference | | | | | |
| **GRADE Working Group grades of evidence** **High certainty:** we are very confident that the true effect lies close to that of the estimate of the effect. **Moderate certainty:** we are moderately confident in the effect estimate: the true effect is likely to be close to the estimate of the effect, but there is a possibility that it is substantially different. **Low certainty:** our confidence in the effect estimate is limited: the true effect may be substantially different from the estimate of the effect. **Very low certainty:** we have very little confidence in the effect estimate: the true effect is likely to be substantially different from the estimate of effect. | | | | | |

#### Explanations

a. Downgraded by two levels due to serious concerns regarding overall risk of bias across the included studies, particularly missing outcome data and limitations in randomization.

b. Downgraded by two levels because the contributing studies had important risk-of-bias concerns, particularly missing outcome data and limitations in the conduct or reporting of the randomized trials.

c. Downgraded by one level because the contributing studies had risk-of-bias concerns, including concerns related to missing outcome data and trial conduct.

d. Downgraded by one level because the 95% CI was wide and crossed the null, and the evidence was based on only 3 studies with 195 participants.

e. Downgraded by one level because the contributing studies had risk-of-bias concerns, including concerns related to missing outcome data and trial conduct.

f. Downgraded by one level because the study estimates showed marked inconsistency (I²=87%), with a wide prediction interval indicating substantial variation in plausible effects across similar settings.

g. Downgraded by one level because only 208 participants from 4 studies contributed to the outcome and the 95% CI was very wide and crossed the null.

h. Downgraded by two levels because the contributing studies had important risk-of-bias concerns, particularly related to missing outcome data and limitations in trial conduct.

i. Downgraded by one level because the study estimates showed inconsistency (I²=57.8%) and the prediction interval indicated substantial uncertainty in the range of plausible effects.

j. Downgraded by one level because the 95% CI crossed the null and included both a potentially beneficial effect and little or no effect.

k. Downgraded by two levels because the contributing studies had important risk-of-bias concerns, particularly related to missing outcome data and limitations in trial conduct.

l. Downgraded by one level because the 95% CI crossed the null and included both a potentially beneficial effect and little or no effect.

m. Downgraded by two levels because the contributing studies had important risk-of-bias concerns, particularly related to missing outcome data and limitations in trial conduct.

n. Downgraded by one level because the 95% CI crossed the null and included both potential benefit and potential harm.

o. Downgraded by one level because only 2 studies with 186 participants contributed to the outcome and the 95% CI was extremely wide and crossed the null.

p. Downgraded by one level because only 180 participants from 5 studies contributed to the outcome and the 95% CI crossed the null.

**Electronic Supplementary Material Appendix S6 (Methodological quality assessment [PEDro])**

| **Author,Year** | **D1** | **D2** | **D3** | **D4** | **D5** | **D6** | **D7** | **D8** | **D9** | **D10** | **D11** | **Total** |
| --- | --- | --- | --- | --- | --- | --- | --- | --- | --- | --- | --- | --- |
| Coknaz et al，2019 | Y | 0 | 0 | 0 | 0 | 0 | 0 | 1 | 1 | 1 | 1 | 4 |
| Wang et al,  2025 | Y | 1 | 1 | 1 | 0 | 0 | 1 | 1 | 0 | 1 | 1 | 7 |
| Comeras-Chueca et al 2022 | Y | 1 | 0 | 1 | 0 | 0 | 0 | 0 | 0 | 1 | 1 | 4 |
| Maddison et al, 2011 | Y | 1 | 1 | 1 | 0 | 0 | 0 | 0 | 1 | 1 | 1 | 6 |
| Murphy et al, 2009 | Y | 1 | 0 | 0 | 0 | 0 | 1 | 1 | 1 | 1 | 1 | 6 |
| Abedelmalek et al, 2022 | Y | 1 | 0 | 1 | 0 | 0 | 0 | 1 | 1 | 1 | 1 | 6 |
| Christison et al, 2016 | Y | 1 | 1 | 1 | 0 | 0 | 0 | 0 | 1 | 1 | 1 | 6 |
| Gao et al,  2019 | Y | 1 | 0 | 1 | 0 | 0 | 1 | 1 | 0 | 1 | 1 | 6 |
| Staiano et al, 2018 | Y | 1 | 1 | 1 | 0 | 0 | 0 | 1 | 0 | 1 | 1 | 6 |
| Staiano et al, 2017 | Y | 1 | 0 | 1 | 0 | 0 | 0 | 1 | 0 | 1 | 1 | 5 |
| Berg et al, 2026 | Y | 1 | 1 | 1 | 0 | 0 | 0 | 0 | 0 | 1 | 1 | 5 |
| Lu et al, 2025 | Y | 1 | 0 | 1 | 0 | 0 | 0 | 0 | 0 | 1 | 1 | 4 |
| Lau et al, 2016 | Y | 1 | 0 | 1 | 0 | 0 | 0 | 0 | 0 | 1 | 1 | 4 |
| Marsigliante et al, 2024 | Y | 1 | 0 | 1 | 0 | 0 | 0 | 1 | 1 | 1 | 1 | 6 |

***Note:*** studies scoring ≥6 are considered **high quality**, those scoring 4-5 are considered **moderate quality**, and those scoring ≤3 are considered **low quality**.

1. eligibility criteria were specified (not included in the total score)

2. subjects were randomly allocated to groups (in a crossover study, subjects were randomly allocated an order in which treatments were received)

3. allocation was concealed

4. the groups were similar at baseline regarding the most important prognostic indicators

5. there was blinding of all subjects

6. there was blinding of all therapists who administered the therapy

7. there was blinding of all assessors who measured at least one key outcome

8. measures of at least one key outcome were obtained from more than 85% of the subjects initially allocated to groups

9. all subjects for whom outcome measures were available received the treatment or control condition as allocated or, where this was not the case, data for at least one key outcome was analysed by “intention to treat”

10. the results of between-group statistical comparisons are reported for at least one key outcome

11. the study provides both point measures and measures of variability for at least one key outcome
